# Neuroprotective Effect of a Multistrain Probiotic Mixture in SOD1^G93A^ Mice by Reducing SOD1 Aggregation and Targeting the Microbiota-Gut-Brain Axis

**DOI:** 10.1007/s12035-024-03988-x

**Published:** 2024-02-13

**Authors:** Zikai Xin, Cheng Xin, Jia Huo, Qi Liu, Hui Dong, Rui Li, Yaling Liu

**Affiliations:** 1 Department of Neurology, The Second Hospital of Hebei Medical University, Shijiazhuang, Hebei People’s Republic of China; 2 The Key Laboratory of Neurology (Hebei Medical University), Ministry of Education, Shijiazhuang, Hebei 050000 People’s Republic of China; 3 Neurological Laboratory of Hebei Province, Shijiazhuang, Hebei 050000 People’s Republic of China

**Keywords:** Amyotrophic lateral sclerosis, Inflammation, Protein aggregation, Autophagy, Enteric nervous system, Microbiota-gut-brain axis

## Abstract

Amyotrophic lateral sclerosis (ALS) is a devastating neurodegenerative disease characterized by the selective loss of motor neurons. A bidirectional communication system known as the “microbiota-gut-brain” axis has a regulatory function in neurodegenerative disorders. The impact of probiotics on ALS through the “microbiota-gut-brain” axis remains uncertain. A longitudinal investigation was conducted to examine the alterations in the structure of the ileum and colon in mutant superoxide dismutase 1 (SOD1^G93A^) transgenic mice models of ALS by using immunofluorescence and Western blotting. Subsequently, the mice were administered a multistrain probiotic mixture (LBE) or vehicle orally, starting from 60 days of age until the terminal stage of the disease. The effects of these agents on the behavior, gut microbiota, microbial metabolites, and pathological processes of the spinal and intestine of SOD1^G93A^ mice were analyzed, with a focus on exploring potential protective mechanisms. SOD1^G93A^ mice exhibit various structural abnormalities in the intestine. Oral administration of LBE improved the proinflammatory response, reduced aberrant superoxide dismutase 1 (SOD1) aggregation, and protected neuronal cells in the intestine and spinal cord of SOD1^G93A^ mice. Furthermore, LBE treatment resulted in a change in intestinal microbiota, an increase in short-chain fatty acid levels, and an enhancement in autophagy flux. SOD1^G93A^ mice exhibited various structural abnormalities in the intestine. LBE can improve the proinflammatory response, reduce aberrant SOD1 aggregation, and protect neuronal cells in the spinal cord and intestine of SOD1^G93A^ mice. The positive effect of LBE can be attributed to increased short-chain fatty acids and enhanced autophagy flux.

**Supplementary Information:**

The online version contains supplementary material available at 10.1007/s12035-024-03988-x.

## Introduction

Amyotrophic lateral sclerosis (ALS) is a devastating neurodegenerative disease characterized by upper and lower motor neuron defects. Its etiology is not yet fully understood. Despite the identification of numerous disease-causing mutations, the understanding of the mechanisms responsible for the development of ALS in most patients is still limited. Similar to other neurodegenerative diseases, pathological protein aggregate within cells is a prominent characteristic of ALS [1]. Over 20 years ago, it was discovered that the aggregated protein of superoxide dismutase 1 (SOD1) was linked to ALS, marking the first association [2]. Since then, this protein has gained significant attention and has become the most extensively researched protein in relation to the development of ALS. Pathological protein aggregates can exert pathologic functions through various mechanisms, including disrupting protein homeostasis, triggering important organelle dysfunction, destroying cell membranes, self-replication, and intercellular transmission [3]. Although several gene mutations can trigger the formation of protein aggregates [4], considerable heterogeneity among patients with the same genotype suggests the involvement of potential environmental and other factors in disease initiation or progression. Currently, clinical and basic research have identified multiple risk factors and disease modifiers, including abnormal metabolism, inflammatory response imbalance, and autophagy impairment [5–7]. While the specific mechanisms underlying the regulation of these factors in the occurrence and progression of ALS remain undefined, several studies have indicated their involvement in the regulation of abnormal protein aggregations [5, 8].

There has been significant attention in the past few years regarding the importance of the structure and function of the intestines in relation to diseases of the nervous system. The gut possesses an extensive neural network, comprising the sympathetic and vagal divisions of the central nervous system (CNS) and the enteric nervous system (ENS). Commonly known as the “second brain,” the ENS is estimated to contain over half a billion neurons [9]. Based on the secretion of a broad spectrum of neurotransmitters, the ENS regulates various intestinal functions and has a close relationship with the intestinal epithelial barrier, the intestinal microbiota, the intestinal immune system, and the CNS [9]. Research has indicated that specific elements that cause neurodegeneration might also play a role in ENS harm, impacting the advancement of diseases and the likelihood of survival [9]. Moreover, the intestine harbors a wide variety of immune cells and provides a crucial mucosal barrier system. The functions of these formations are crucial in inhibiting the translocation of disease-causing microorganisms into the body, as well as regulating both local and systemic inflammatory responses [10–12]. The existing evidence indicates that various neurological disorders are linked to dysfunctional tight junctions and an imbalanced relationship between pro-inflammatory and anti-inflammatory processes in the intestines [11, 13, 14], suggesting that intestinal structural abnormalities may play certain roles in regulating the progression of ALS.

The intestine is inhabited by an enormous number of microorganisms. It is anticipated that there would be a proportion of roughly 1:1 in relation to the total count of human cells [15]. According to the present research, the gut microbiome contributes to the pathophysiology of the CNS. This effect can be ascribed to the “microbiota-gut-brain” axis. Research indicates that the intestinal microbiome has the potential to impact the CNS through various pathways, including the neuroactive, immune, neural, and endocrine pathways, or producing small molecular metabolites, like short-chain fatty acids (SCFAs) [16, 17]. Sampson and colleagues [18] observed that the depletion of the gut microbiota through antibiotics resulted in reduced microglial activation, decreased aSyn inclusions, and fewer mobility issues in mice that over-expressed human α-Synuclein (aSyn). Multiple research studies have shown that the administration of particular microorganisms, like *Akkermansia muciniphila*, or substances produced by the microbiota, such as nicotinamide and butyrate, may enhance motor function and reduce pathological alterations in the spinal cord and intestine of SOD1^G93A^ mice [19, 20]. According to a study conducted on mice with chromosome 9 open reading frame 72 (C9orf72) loss of function (LOF) mutations, it was found that the gut microbiome has a strong correlation with both systemic and CNS inflammatory characteristics [21]. These researches have indicated the promising possibilities of regulating microbiota in the treatment of ALS.

Probiotics can effectively modulate intestinal microbiota composition or metabolic/immunological activity [22, 23]. As a readily available biological product with a long history, probiotics have a favorable safety profile and convenience. Recent researchers have suggested that probiotics can regulate intestinal microbiota, affect mucosal barrier function, generate immunomodulatory properties, and produce various advantageous health outcomes [24–26]. Moreover, probiotic supplementation typically leads to an increase in SCFAs in both feces and serum, as observed in previous studies [24, 27–29]. These microbial byproducts can control various metabolic pathways in the gut and other organs like the liver, muscle, and brain, including the liver, muscle, and brain, and have a positive impact on health by regulating energy metabolism and homeostasis, inflammation, immunity, and multiple aspects [30]. Among bacterial strains exhibiting the aforementioned characteristics, several belong to the species *Lactobacillus acidophilus* [31–35], *Bifidobacterium longum* [31–33, 35, 36], and *Enterococcus faecalis* [37–39]. Notably, in several neurodegenerative diseases, specific probiotics have been reported to regulate intestinal microbiota, inhibit microglial activation and neuroinflammation, reduce the accumulation of aberrant proteins, and attenuate disease progression [40–44]. The results indicate that probiotics could potentially provide new and innovative treatment approaches for ALS. Thus, we assume that a commercialized multistrain probiotic preparation that is a blend of three strains belonging to *Lactobacillus acidophilus*, *Bifidobacterium longum*, and *Enterococcus faecalis* (abbreviated as LBE in the current study) has the potential to modulate the intestinal microbiota, ameliorate intestine and spinal cord pathology, and extend survival time in ALS.

In this study, we employed ALS transgenic mice harboring mutant superoxide dismutase 1 (SOD1^G93A^) to examine this hypothesis. A longitudinal investigation was carried out to examine the alterations in the structure of the ileum and colon in SOD1^G93A^ mice at the ages of 40, 90, and 120 days. In order to investigate the healing properties of LBE in ALS, we administered LBE or vehicle orally to SOD1^G93A^ mice starting at 60 days old until the terminal phase of disease. We examined the impact of these substances on the behavior and pathological processes of the spinal and intestinal of SOD1^G93A^ mice. Finally, we examined the influences of LBE on gut microbiota and serum SCFAs and explored the possible protective mechanisms. We found that SOD1^G93A^ mice exhibited various structural abnormalities in the intestine. The utilization of LBE can improve the proinflammatory response, reduce aberrant SOD1 aggregation, and protect neuronal cells in the spinal cord and intestine of SOD1^G93A^ mice. The positive effect of LBE can be attributed to increased SCFAs and enhanced autophagy flux.

## Materials and Methods

### Animal Model

Jackson Laboratory (Bar Harbor, ME, USA) provided us with SOD1^G93A^ transgenic mice. The transgenic mice were bred with wild-type (WT) B6SJL/F1 females to produce hemizygotes and maintain them. Polymerase chain reaction (PCR) was used for genotyping the mice. All experiments were performed using mice of the same age and sex, and their WT littermates served as controls. The mice were housed in a controlled environment with alternating 12-h periods of light and darkness, maintaining a consistent room temperature of 22 °C ± 2 °C. They were provided with sterilized food and water. All procedures involving animals were conducted in compliance with the Regulations for the Administration of Laboratory Animals in China. All experiments were approved by the Research Ethics Committee of the Second Hospital of Hebei Medical University (Approval No. 2022AE269).

### Animal Experiment Grouping and Dosing Regimen

**Fig. 1:**
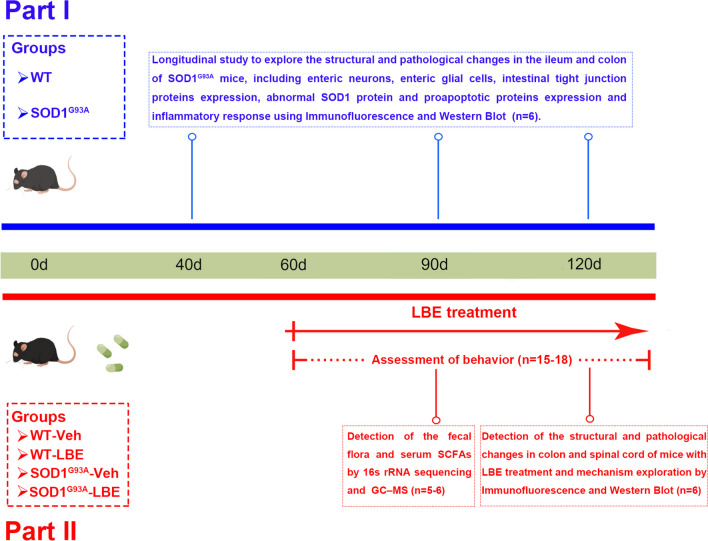
The graphical timeline for animal experiments. The blue line represents the experimental timeline, including the SOD1^G93A^ and WT groups. The red line corresponds to the experimental timeline for four groups (WT-Veh, WT-LBE, G93A-Veh, and G93A-LBE) of mice (*n* represents the number of animals in each group)

This research was divided into two parts. In the first part, a longitudinal study was conducted in order to investigate the structural and pathological changes in the ileum and colon of SOD1^G93A^ mice at the ages of 40, 90, and 120 days. During this phase of the study, the mice were categorized into two groups: SOD1^G93A^ and WT groups. The therapeutic impact of LBE in ALS was investigated in the second segment. Two groups were formed by randomly dividing SOD1^G93A^ mice, namely the LBE- and vehicle-treated groups. Age- and gender-matched WT littermate mice served as controls and were also randomly divided into LBE- and vehicle-treated groups. The experimental group was administered the LBE capsule containing live bacteria of *Lactobacillus acidophilus*, *Bifidobacterium longum*, and *Enterococcus faecalis*, which was obtained from Shanghai Xinyi Pharmaceutical Co. Ltd., China. LBE (one capsule = 210 mg) was suspended in physiological saline (600 µl) to prepare live bacterial suspensions. From postnatal day 60 until the terminal phase of disease, the treatment group received a daily oral gavage of 300 µl of live bacterial suspensions. A sample size of 15 to 18 female and male mice in each group was arbitrarily chosen to assess motor function, disease onset, and survival time. Other female mice were killed at 40, 90, and 120 days for immunofluorescence, Western blotting analysis, 16S rDNA sequencing, and gas chromatography-mass spectrometry (GC‒MS). Figure 1 depicts the experimental timeline.

### Assessment of Behavior in Mice

The behaviors of the SOD1^G93A^ mice were evaluated using body weight, step length, performance on the rotarod test, and neurological scoring [20, 45]. Body weight was assessed twice a week beginning at 60 days old. The step length, performance on the rotarod test, and neurological scoring were assessed weekly starting at 80 days of age, and were evaluated twice a week from 120 days of age until the endpoint. Behavioral experiments took place from 7 to 9 p.m.

#### Rotarod Locomotor Test

Motor coordination was assessed using the rotarod locomotor test. The mice were positioned on a spinning rod and the speed gradually increased from 2 to 30 rpm within a span of 3 min in a rotating rod apparatus (Ugo Basile, Italy). The apparatus automatically measured the time each mouse lost balance and fell off the rod. Prior to the official locomotor examination, the mice were typically accustomed to the rotating rod device through training. Three repetitions of each experiment were carried out, with a 30-min interval between each repetition, and the longest attempt was documented.

#### Neurological Scoring

Scoring of the mice was conducted according to the criteria [46]. When the mice were suspended by the tail and the hindlimbs presented full extension and the mice could maintain the posture for at least 2 s, the score was 0. When the mice were suspended by the tail and their hindlimbs exhibited abnormal splays, e.g., collapse, partial collapse toward the lateral midline, trembling and retracting, or a clasp of hind legs, the score was 1. When the toes were flexed downwards at least two times while walking 90 cm, or any part of the foot scraped against the floor/table during locomotion, the score was 2. When there was severe immobility or limited mobility in the hindlimbs, the score was 3. When the mice could not right themselves on their side within 30 s, the score was 4.

#### The Onset of Disease and Survival [20, 45]

Disease onset occurs when the mice are unable to endure three rotarod locomotor tests for a minimum of 3 min and/or achieve a neurological score of 1. The mice were observed daily starting from 120 days old. The final phase of SOD1^G93A^ mice was determined upon reaching a neurological rating of 4 and/or experiencing a loss of over 15% of their total body weight.

### Immunofluorescence

The mice were given sodium pentobarbital (60 mg/kg, i.p.) to induce anesthesia. The subject was then perfused with ice-cold phosphate-buffered saline (PBS, pH 7.4) through the transcardial route. The lumbar spinal cord (L4–L5), ileum, and colon were extracted and preserved in paraformaldehyde for 12 h under cold temperatures. These tissues were dehydrated, embedded, and sliced. Immunofluorescence staining was performed as described before [36]. Antibodies used are as follows: IL-1β (1:500, Santa Cruz Biotechnology, sc-52012), ChAT (1:100, EMD Millipore, AB144P), NeuN (1: 600, NOVUS, NBP1-92,693), MAP2 (1:2000, BOSTER, M01201-3), SOD1 (1:200, Abcam, ab16831), IBA1 (1: 500, Abcam, ab178847), casepase3 (1:200, Affinity, AF7022), GFAP (1:200, Cell Signaling, # 3670), Claudin1 (1:200, Proteintech, 13050–1-AP), PGP9.5 (1:400, HUABIO, ET1703-22), TNF-α (1: 200, Santa Cruz Biotechnology, sc-52746), LC3 (1:500, Sigma, L7543), and p62 (1:500, Abcam, AB56416). All secondary antibodies were obtained from Invitrogen and Abcam. DAPI Fluoromount-G (Southern Biotech) was used to stain the nucleus. The segments were observed using a fluorescence confocal microscope (LSM900, ZEISS, Germany).

To perform whole-mount immunofluorescence staining, the ileum and colon were extracted, cleansed, flattened, and fixed in paraformaldehyde for 12 h under low-temperature circumstances. After delicately scraping the mucosal layer using a scalpel, the intestinal tissues obtained were subsequently sliced into squares measuring 0.5 cm × 0.5 cm. The tissues were treated with PBS-0.3% Triton X-100 for 60 min to make the tissue permeable. Subsequently, the tissues were blocked in PBS containing 10% goat serum at a temperature of 4 °C for a duration of 48 h. After that, the tissues were incubated with PGP9.5 (1:400, HUABIO, ET1703-22), GFAP (1:200, Cell Signaling, # 3670), and TNF-α (1 200, Santa Cruz Biotechnology, sc-52746) at a temperature of 4 °C for a duration of 72 h. Following washing with 1 × PBS, 12 times for 60 min each, the slices were exposed to secondary antibodies for 48 h at 4 °C. Subsequently, the slices were washed with 1 × PBS for 12 cycles lasting 60 min each. The segments were observed using a fluorescence confocal microscope (LSM900, ZEISS, Germany).

### Western Blot Analysis

The lumbar spinal cord, ileum, and colon were collected. The protein extraction and Western blotting (WB) were performed according to the previous method [36]. Antibodies used are as follows: IL-10 (1:500, Immunoway, #YT5138), TNF-α (1:200, Santa Cruz Biotechnology, sc-52746), GFAP (1:200, Cell Signaling, #3670), SOD1 (1:200, Abcam, ab16831), Claudin1 (1:500, Invitrogen, 37–4900), Occludin (1:100, Invitrogen, 71–1500), IL-1β (1:500, Santa Cruz Biotechnology, sc-52012), LC3 (1:500, Sigma, L7543), and p62 (1:1000, Sigma, P0067). GAPDH (1:5000, Proteintech, 1049–1-AP) or β-actin (1:1000, Proteintech, 60008-1) was used as an internal control. Acquisition of images was done using Odyssey Infrared Imaging System (LI-COR, Lincoln, NE, USA). ImageJ was utilized to quantify the intensities of the bands.

### 16S rDNA Sequencing

The ceca and their contents from every mouse were removed and rapidly frozen in liquid nitrogen to be stored at a temperature of − 80 °C. To analyze the composition of cecal microbiota in different groups, we performed 16S rDNA sequencing at Shanghai Zhongke New Life Biotechnology Co. The extraction of fecal genomic DNA was carried out using the fecal microbiota DNA extraction kit (DP712) in accordance with the guidelines provided by the manufacturer. The PCR was conducted using the Phusion® High-Fidelity PCR Master Mix with GC Buffer from New England Biolabs. The TruSeq® DNA PCR-Free Sample Preparation Kit (Illumina) was utilized for library construction. All PCR amplification of microbial samples was analyzed using the Illumina NovaSeq6000 platform (Illumina). Uparse (http //drive5.com/uparse/) was used to cluster the clean reads of all samples into operational taxonomic units (OTUs), with a 97% identity threshold. The Silva database (http://www.arb-silva.de/) was used to perform species annotation on the representative sequence of the OTU. The α-diversity of the community was determined by utilizing the Chao1 and ACE indices.

### Determination of Serum SCFA Concentrations

After the mice had fasted for 3 h, eye blood samples were collected using coagulation-promoting tubes. The samples were then centrifuged at a speed of 3000 rpm for 10 min to acquire serum. The serum SCFA concentration was determined by Shanghai Zhongke New Life Biotechnology Co. Sample separation was carried out using an ADB-Wax column (30 m × 0.25 mm ID × 0.25 μm; Agilent Technologies Inc.). GC‒MS detector (Agilent 7890A/5975C, USA) was utilized for GC‒MS analysis. The retention time and peak area of chromatography were extracted using MSD ChemStation software. The concentration of each SCFA in the sample was calculated using a standard curve generated from standard samples.

### Statistical Analysis

The statistical analysis was conducted utilizing the SPSS software (SPSS version 21.0). All data are expressed as the mean ± SEM. Differences between the two groups were assessed using the *t* test. Differences among more than two groups were assessed using repeated-measures analysis of variance (ANOVA) followed by Tukey or LSD post hoc tests for multiple comparisons. Values of *p* < 0.05 were considered statistically significant. The probability of survival was calculated using the Kaplan–Meier method, and statistical analysis was performed using a log-rank test.

## Results

### Intestinal Barrier Impairment and Inflammatory Activation in SOD1^G93A^ Mice

**Fig. 2:**
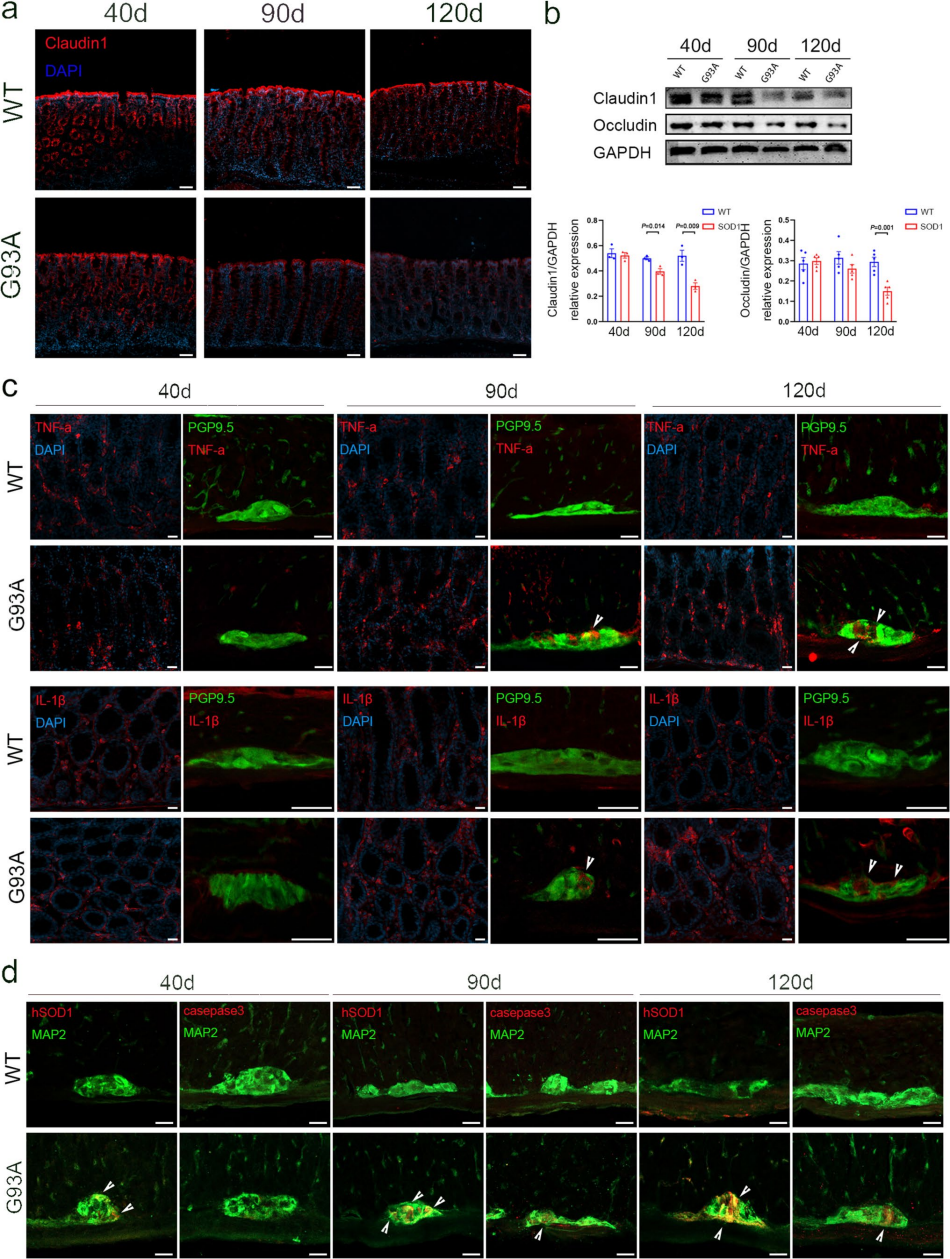
Structural alterations in the colon of SOD1^G93A^ mice. **a** Immunofluorescence labeling for Claudin1 (red) in the colon of SOD1^G93A^ and WT mice at 40 days, 90 days, and 120 days of age. Scale bar = 50 μm. **b** Western blot analysis of Claudin1 and Occludin levels in the colon of SOD1^G93A^ and WT mice at 40 days, 90 days, and 120 days of age. Quantification of Claudin1 and Occludin relative to GAPDH (*n* = 3–5 mice per group). **c** Immunofluorescence staining for TNF-α (red) and IL-1β (red) in the mucosa and co-immunofluorescence staining of TNF-α (red) and PGP9.5 (green), IL-1β (red) and PGP9.5 (green) in the muscle layer of the colon of SOD1^G93A^ and WT mice at 40 days, 90 days, and 120 days. White arrowheads show the portions of TNF-α^ +^ and IL-1β^ +^ . Scale bar = 20 μm. **d** Co-immunofluorescence staining of hSOD1 (red) and MAP2 (green), casepase3 (red) and MAP2 (green) in the muscle layer of the colon of SOD1^G93A^ and WT mice at 40 days, 90 days, and 120 days of age. White arrowheads show the portions of hSOD1^ +^ and casepase3^ +^ . Scale bar = 20 μm. Nuclei were stained with DAPI (blue)

We first detected the expression of tight junction (TJ) proteins in the intestine of SOD1^G93A^ mice, including Occludin and Claudin1. These proteins play a crucial role in preventing the entry of intestinal harmful contents, thereby acting as a vital protective barrier for the organism. In the colon of 90-day-old SOD1^G93A^ mice, we observed a reduction in the levels of claudin1 and occludin expression, which became more pronounced at 120 days (Fig. 2a and b). Given the correlation between disturbance of the intestinal epithelial barrier and both intestinal inflammation and systemic inflammation [11], our investigation also encompassed the examination of inflammation in the intestines of SOD1^G93A^ mice. Immunofluorescence staining demonstrated that the positive staining of proinflammatory cytokines, including tumor necrosis factor-α (TNF-α) and interleukin-1β (IL-1β), accumulated primarily on the intestinal mucosal layer and myenteric ganglia (labeled with panneuronal marker PGP9.5) of SOD1^G93A^ mice (data not shown). In comparison to WT littermate controls, we observed an increased expression of TNF-α and IL-1β in the colonic mucosal layer and myenteric ganglia of 90-day-old SOD1^G93A^ mice, which became even more pronounced in 120-day-old SOD1^G93A^ mice (Fig. 2c). Nevertheless, we noticed minimal or absence of reduced expression of TJ proteins in the ileum during this period (Fig. S1a and b). In addition, we observed that the levels of TNF-α and IL-1β were elevated solely in the myenteric ganglia of the ileum in 120-day-old SOD1^G93A^ mice when compared to those in WT littermate controls (Fig. S1c).

A preliminary examination was carried out on the ENS of mice with the SOD1^G93A^ mutation. Through the utilization of microtubule-associated protein 2 (MAP2) (pan-neuronal marker) and mutant human SOD1 (hSOD1) immunofluorescence staining, it was discovered that mutant SOD1 protein aggregates in the ENS of SOD1^G93A^ mice, particularly in the myenteric ganglia (data not shown). In a longitudinal investigation, it was found that there was an abnormal accumulation of SOD1 in the myenteric ganglia of SOD1^G93A^ mice at 40 days old, and this accumulation steadily grew as the disease advanced (Fig. 2d). Abnormal protein aggregates typically result in abnormal cell structure and function or cell death. In the following step, we investigated the presence of the proapoptotic protein casepase3 and observed a rise trend in its quantity as the aggregates of mutant SOD1 protein increased (Fig. 2d). Notably, aberrant SOD1 aggregation was also observed in the ileum (Fig. S1d), but the degree of the pathological change was milder than that in the colon (Fig. S1e).

### Massive Loss of Enteric Myenteric Neurons in SOD1^G93A^ Mice

**Fig. 3:**
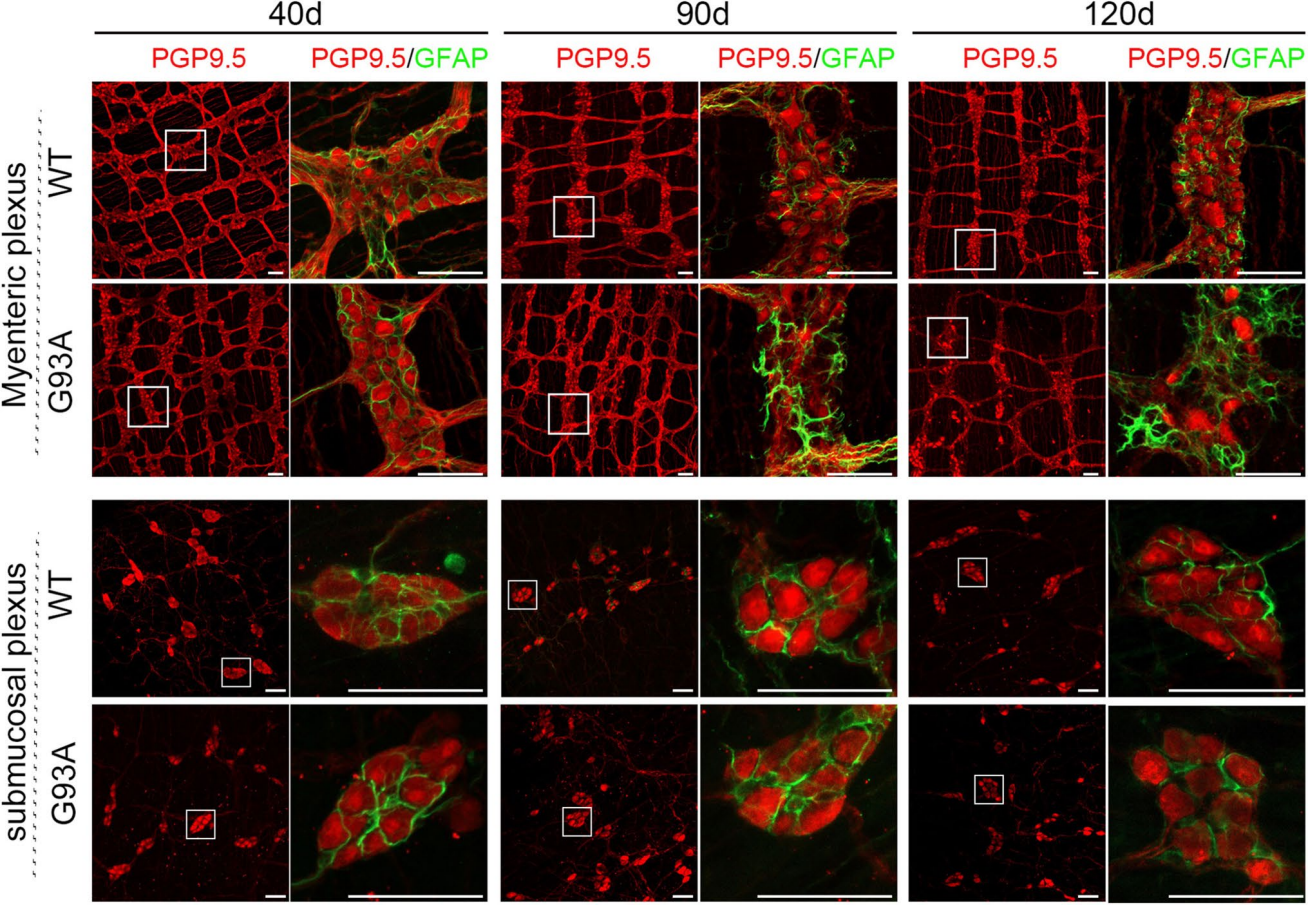
Whole-mount immunofluorescence staining of the ENS showed massive loss of enteric myenteric neurons in the colon of SOD1^G93A^ mice. Co-immunofluorescence staining of PGP9.5 (red) and GFAP (green) in the myenteric plexus and submucosal plexus of the colon of SOD1^G93A^ and WT mice at ages of 40, 90, and 120 days. Scale bar = 50 μm

We performed intestinal whole-mount immunofluorescence staining with PGP9.5 and glial fibrillary acidic protein (GFAP)—an enteric glial cell (EGC) marker. At 40 days of age, we noticed a disruption in the EGC network of the myenteric plexus in the colon of SOD1^G93A^ mice, when compared to littermate WT mice (Fig. 3). Furthermore, the 90-day-old SOD1^G93A^ mice exhibited neuronal loss and glial cell proliferation in the myenteric plexus of both the colon and ileum, which was more significant in 120-day-old mice (Figs. 3 and S2). However, this anomalous phenomenon is not evident in the submucosal plexus (Figs. 3 and S2).

### LBE Improved Enteric Injury in SOD1^G93A^ Mice

**Fig. 4:**
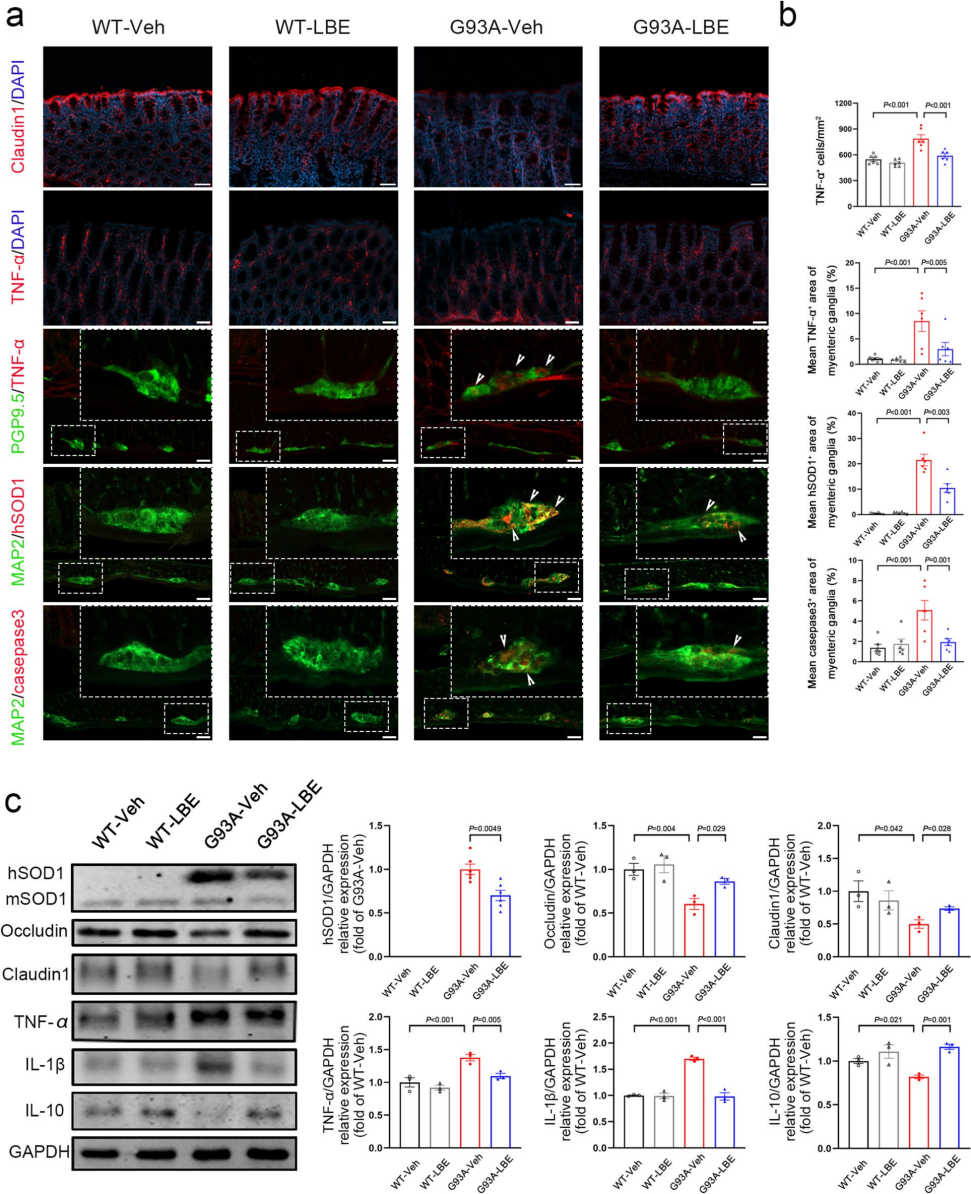
LBE improved enteric injury in SOD1^G93A^ mice. **a** Immunofluorescence staining of Claudin1 (red) in the colon of mice in all groups at 120 days of age; immunofluorescence labeling for TNF-α (red) in the mucosa of the colon of mice in all groups at 120 days of age; co-immunofluorescence staining of TNF-α (red) and PGP9.5 (green), hSOD1 (red) and MAP2 (green), and casepase3 (red) and MAP2 (green) in the muscle layer of the colon of mice in all groups at 120 days of age. Scale bar = 50 μm. White arrowheads show the portions of TNF-α ^+^ , hSOD1 ^+^ , and casepase3 ^+^ . **b** Quantification of the number of TNF-α^ +^ cells per mm^2^ in the mucosa of the colon. Percentage analysis of TNF-α ^+^ , hSOD1 ^+^ , and casepase3 ^+^ areas in the myenteric ganglia. **c** Western blot analysis of SOD1, Occludin, Claudin1, TNF-α, IL-1β, and IL-10 levels in the colon of mice in all groups at 120 days of age. Quantification of SOD1, Occludin, Claudin1, TNF-α, IL-1β, and IL-10 relative to GAPDH. *n* = 3–6 mice per group. Nuclei were stained with DAPI (blue)

**Fig. 5:**
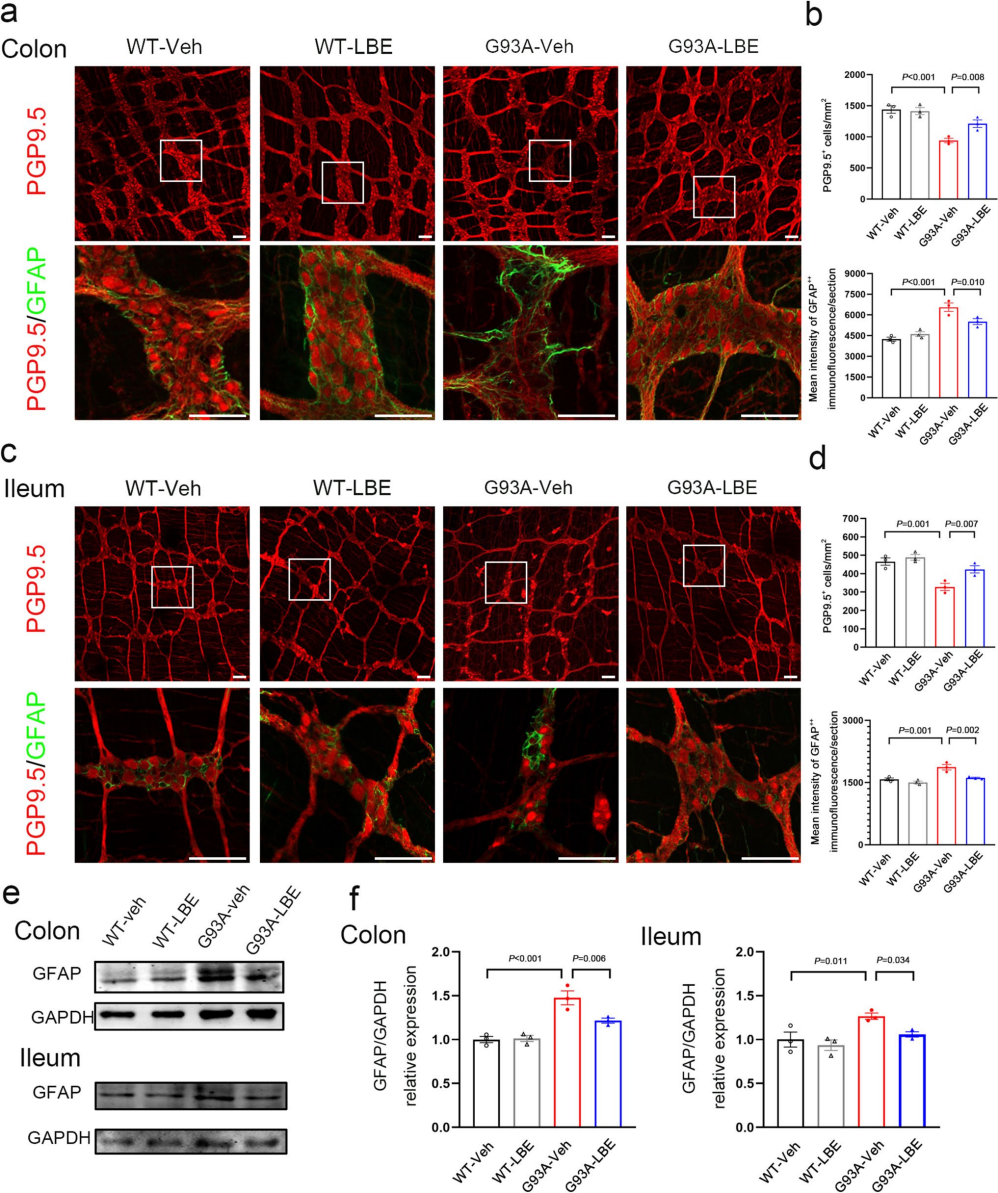
Whole-mount immunofluorescence staining of the ENS showed that LBE increased the number of enteric myenteric neurons in SOD1^G93A^ mice. **a** Co-immunofluorescence staining of PGP9.5 (red) and GFAP (green) in the myenteric plexus of the colon of SOD1^G93A^ and WT mice treated with or without LBE at 120 days. Scale bar = 50 μm. **b** Quantification of the number of PGP9.5 ^+^ cells per mm^2^ in the myenteric plexus of the colon. Quantification of the intensity of GFAP ^+^ immunofluorescence in the myenteric plexus of the colon. **c** Co-immunofluorescence staining of PGP9.5 (red) and GFAP (green) in the myenteric plexus of the ileum of SOD1^G93A^ and WT mice treated with or without LBE at 120 days of age. Scale bar = 50 μm. **d** Quantification of the number of PGP9.5 ^+^ cells per mm^2^ in the myenteric plexus of the ileum. Quantification of the intensity of GFAP ^+^ immunofluorescence in the myenteric plexus of the ileum. **e** Western blot analysis of GFAP levels in the colon and ileum of mice in all groups at 120 days of age. **f** Quantification of GFAP relative to GAPDH. *n* = 3 mice per group

**Fig. 6:**
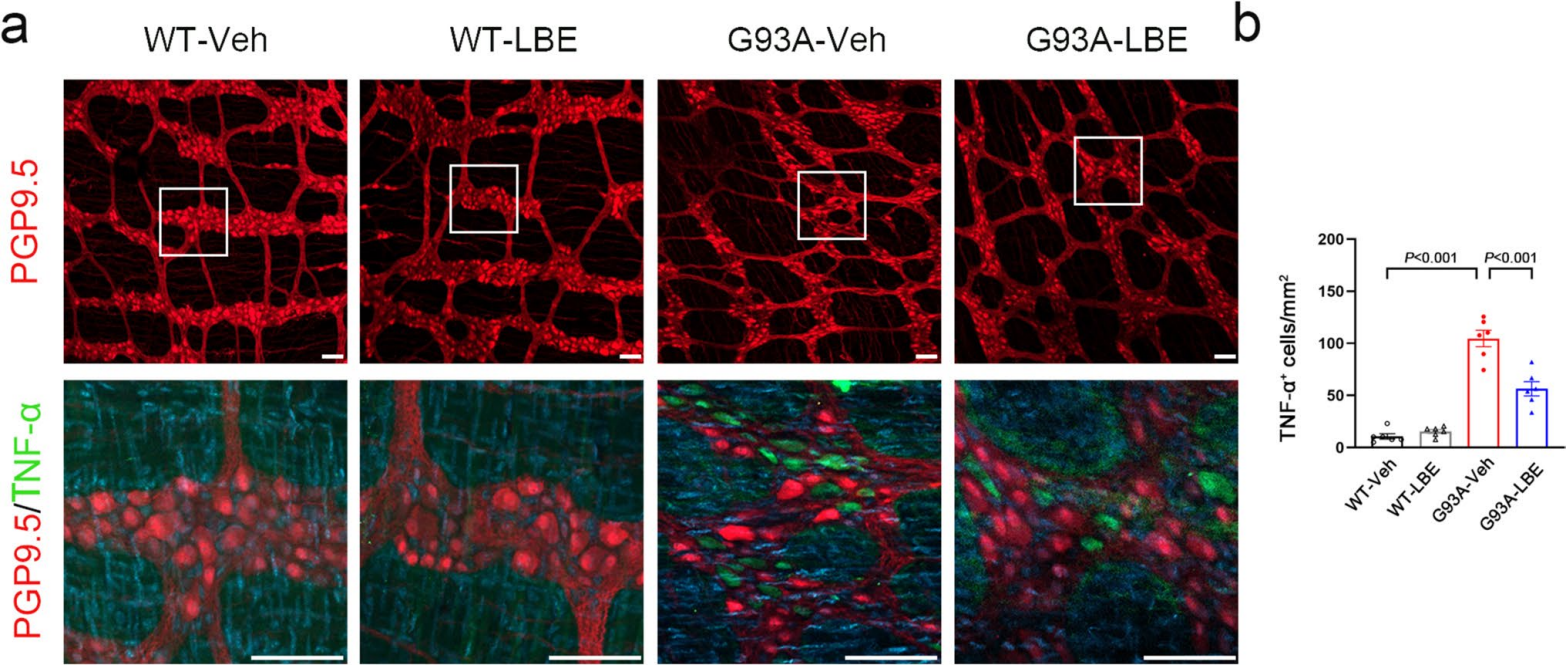
Whole-mount immunofluorescence staining of the ENS showed that LBE attenuated inflammatory activation in the myenteric ganglia of SOD1^G93A^ mice. **a** Colocalization of PGP9.5 (red) and TNF-α (green) in the myenteric ganglia of the colon of SOD1^G93A^ and WT mice treated with or without LBE at 120 days of age (scale bar = 50 μm). **b** Quantification of the number of TNF-α ^+^ cells per mm^2^ in the myenteric plexus of the colon. (*n* = 2 independent experiments with three mice per group)

To verify the effect of LBE on intestinal pathology, the mice were divided into four groups in a random manner. The treatment group received LBE from postnatal day 60 to the end-points, while the control mice received vehicle. After 60 days of initial treatment, immunofluorescence staining was conducted on colon sections from all groups. The results revealed that the administration of LBE led to an enhanced expression of Claudin1 (Fig. 4a and b). Furthermore, the G93A-LBE mice exhibited a significant downregulation of TNF-α in the colonic mucosal layer and myenteric ganglia, as indicated by immunostaining for TNF-α and PGP9.5, in comparison to G93A-Veh mice (Fig. 4a and b). Moreover, LBE therapy decreased the levels of casepase3 and SOD1 aggregates in the myenteric ganglia (Fig. 4a and b). Nevertheless, there was no notable difference in WT-LBE compared to WT-Veh (Fig. 4a and b). In G93A-Veh mice, the WB results revealed that the expression of Claudin1, Occludin, and interleukin-10 (IL-10) was downregulated; that of hSOD1, TNF-α, and IL-1β was upregulated compared to WT-Veh mice. However, treatment with LBE reversed this tendency (Fig. 4c). In order to gain a deeper comprehension of the impact of LBE treatment on the ENS, we performed whole-mount staining. As shown in Fig. 5, the administration of LBE resulted in an increase in the quantity of PGP9.5-positive cells and a decrease in the activation of glial cells in the myenteric plexus of both the colon and ileum (Fig. 5a–d). WB results also demonstrated that the levels of GFAP were increased in the colon and ileum of SOD1^G93A^ mice when compared to WT littermates. However, treatment with LBE resulted in a decrease in GFAP expression (Fig. 5e and f). Furthermore, whole-mount immunostaining for TNF-α and PGP9.5 revealed that, compared to littermate WT controls, the number of TNF-α-positive cells in the myenteric ganglia increased in SOD1^G93A^ mice. This phenomenon was reversed using LBE therapy, but there were no significant differences between WT-LBE and WT-Veh (Fig. 6). Together, these findings indicate that LBE treatment improved enteric injury in SOD1^G93A^ mice.

### LBE Reduced the Accumulation of Abnormal SOD1 Protein and Increased the Number of Neurons in the Spinal Cord of SOD1^G93A^ Mice

**Fig. 7:**
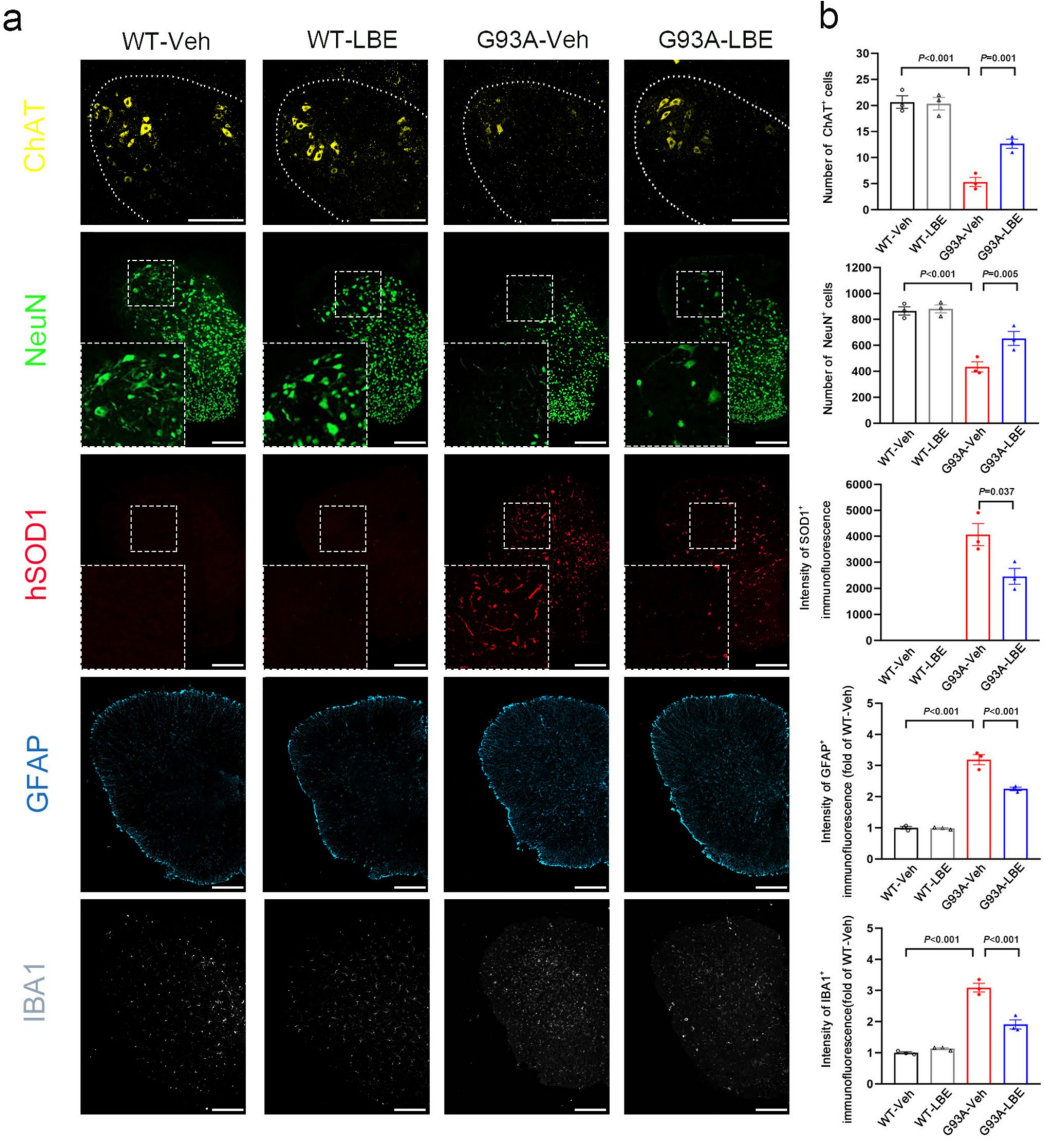
LBE reduced the accumulation of abnormal SOD1 protein and increased the number of neurons in the spinal cord of SOD1^G93A^ mice. **a** Immunofluorescence labeling for ChAT (yellow), NeuN (green), hSOD1 (red), GFAP (blue), and IBA1 (gray) in the lumbar spinal cord of mice in all groups at 120 days. Scale bar = 200 μm. **b** Quantification of ChAT ^+^ cells, NeuN ^+^ cells, SOD1 intensity, GFAP intensity, and Iba1 intensity. *n* = 3 mice per group

In order to assess the impact of LBE on the survival of motor neurons (MN), immunofluorescence staining of choline acetyltransferase (ChAT) was conducted in the L4–5 spinal cord of mice in each group. The findings showed a significant loss of MN in SOD1^G93A^ mice at 120 days old, while treatment with LBE enhanced the survival of motor neurons (Fig. 7a and b). Following that, we measured the number of neurons in the lumbar spinal cord by neuronal nuclei (NeuN) immunofluorescence staining. It was observed that the number of neurons was considerably greater after LBE treatment compared to vehicle treatment in SOD1^G93A^ mice (Fig. 7a and b). Furthermore, compared with vehicle treatment, LBE treatment resulted in a reduction of abnormal SOD1 aggregation in the spinal cord of SOD1^G93A^ mice at 120 days of age (Fig. 7a and b). Notably, astrocyte and microglia overactivation is also correlated with disease progression. Consequently, we examined their expression through immunofluorescence and observed a significant decrease in microglia and astrocyte proliferation in G93A-LBE mice at 120 days old when compared to G93A-Veh mice (Fig. 7a and b). In short, these results indicated that LBE therapy mitigated spinal cord pathological alterations in SOD1^G93A^ mice.

### LBE Alleviated Motor Deficits and Increased the Lifespan of SOD1^G93A^ Mice

**Fig. 8:**
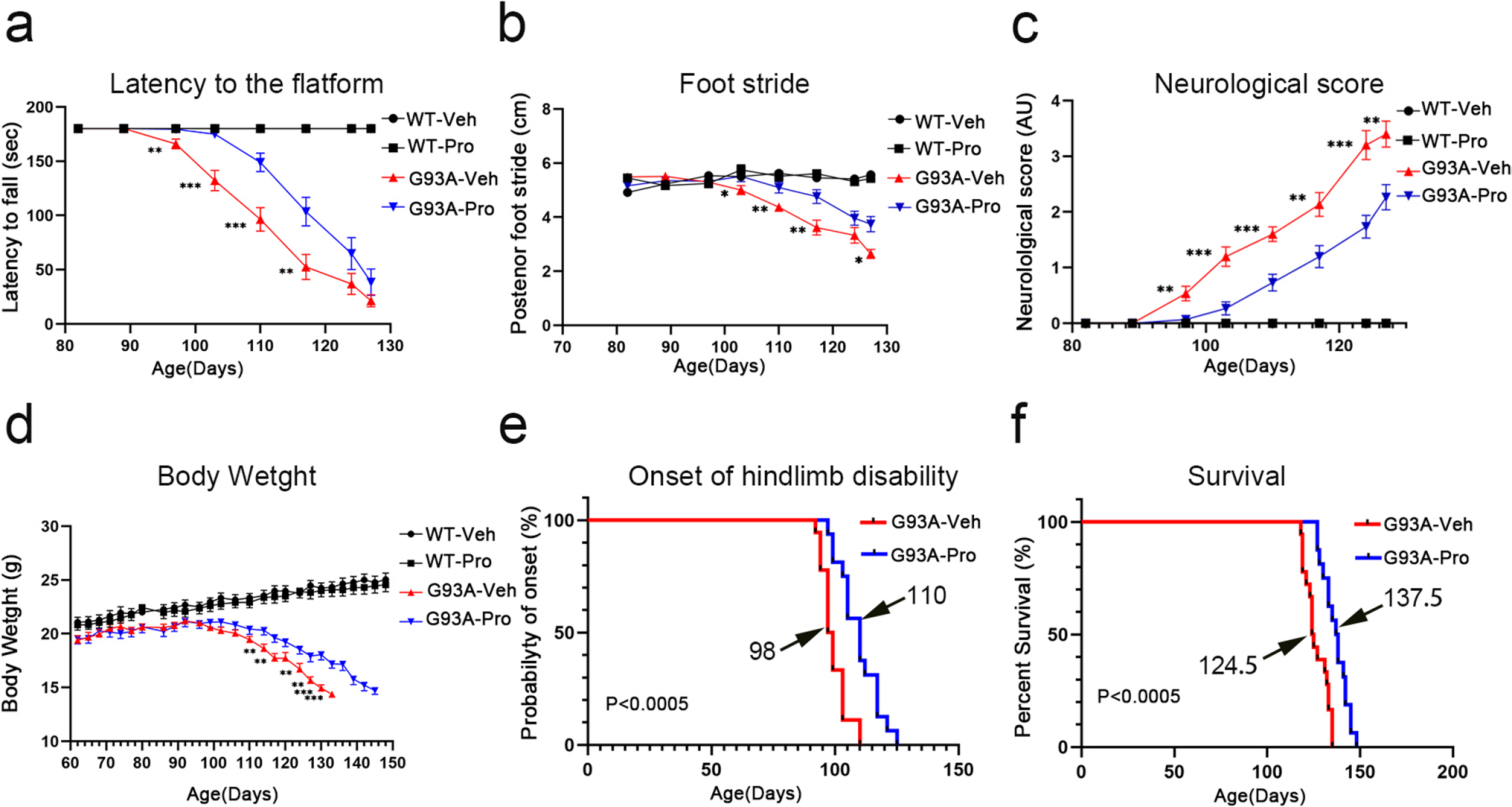
LBE ameliorated motor degeneration and increased lifespan in SOD1^G93A^ mice. **a**–**c** Assessment of behavior of mice in all groups by the rotarod locomotor test (**a**), the foot stride test (**b**), and neurological scoring (**c**). **d** The weight curves of mice (WT-Veh, WT-LBE, G93A-Veh, and G93A-LBE mice). **e**–**f** Probability of disease onset and survival of vehicle- and LBE-treated SOD1^G93A^ mice. (*n* = 15–18 mice per group, *P < 0.05, **P < 0.01, ***P < 0.001 as compared with G93A-Veh group)

Subsequently, we monitored the effects of LBE on the motor function of SOD1^G93A^ mice. As quantified by the rotarod locomotor test, neurological scoring, and foot stride test, we found that G93A-LBE mice had significantly better motor function compared to the G93A-Veh mice, and LBE had no effect on WT mice (Fig. 8a–c). As the decline in body weight also serves as a clinical indicator for disease progression in this model, we observed G93A-LBE mice and discovered that these mice experienced a gradual reduction in body weight, while G93A-Veh mice exhibited rapid weight loss throughout the progression of the disease. However, neither WT-LBE nor WT-Veh mice demonstrated any weight loss (Fig. 8d). Notably, compared with vehicle treatment, LBE treatment significantly prolonged the onset of hindlimb disability (median, 98 days vs. 110 days; *P* = 0.0004) and lifespan (median, 124.5 days vs. 137.5 days; *P* = 0.0005) of SOD1^G93A^ mice (Fig. 8e and f). Altogether, the LBE-treated SOD1^G93A^ mice exhibited relatively better locomotor function and longer survival time than the vehicle-treated mice.

### LBE Treatment Modulated the Intestinal Microbiota Composition of SOD1^G93A^ Mice

**Fig. 9:**
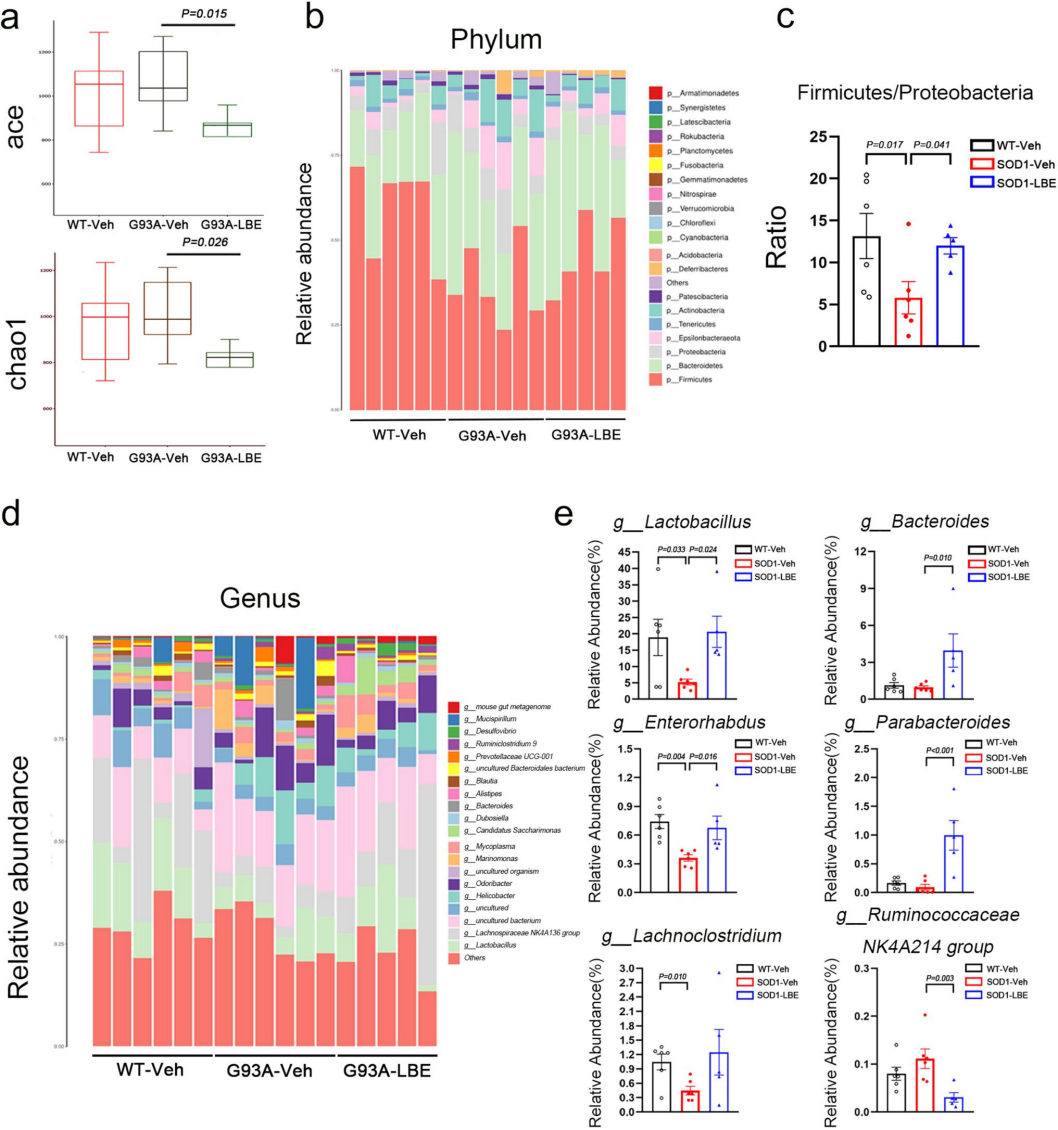
LBE modulated gut microbiota composition in SOD1^G93A^ mice. **a** Comparison of the ACE index and Chao index based on OTU levels among different groups (WT-Veh, G93A-Veh, and G93A-LBE mice). **b** Relative abundances of the bacterial genera in the fecal specimens at the phylum level among different groups (WT-Veh, G93A-Veh, and G93A-LBE mice). **c** The ratio of Firmicutes to Proteobacteria. **d** Relative abundances of bacterial genera in fecal specimens at the genus level among different groups (WT-Veh, G93A-Veh, and G93A-LBE mice). **e** Quantification of the relative abundances of *Lactobacillus*, *Bacteroides*, *Enterorhabdus*, *Parabacteroides*, *Lachnoclostridium*, and the *Ruminococcaceae NK4A214 group*. *n* = 5–6 mice per group

In order to study the impact of LBE treatment on the gut microbiome of SOD1^G93A^ mice, we utilized 16S rDNA sequencing to analyze the taxonomic makeup of the gut microbiome after administering LBE therapy to SOD1^G93A^ mice. As demonstrated in Fig. 9a, there was no noticeable distinction in alpha diversity, as determined by the ACE and Chao1 indexes, between the SOD1^G93A^ mice and WT littermate controls. Nevertheless, the LBE therapy resulted in a decrease in alpha diversity. Furthermore, we investigated the relative abundance of the microbiota of each group and identified several potentially disease-related microbiotas. The phylum-level distribution of bacteria for each group is depicted in Fig. 9b. In all groups, we noticed that the most prevalent phyla were Firmicutes, Bacteroidetes, Bacteroidetes, Epsilonbacteraeota, and Tenericutes. Compared with the WT littermate control, the SOD1^G93A^ mice had a significantly decreased Firmicutes to Proteobacteria ratio, which was reversed by LBE treatment (Fig. 9b and c). At the genus level, we observed a significant decrease in the proportions of *Lactobacillus*, *Enterorhabdus*, and *Lachnoclostridium* in SOD1^G93A^ mice when compared to WT control mice. The LBE treatment significantly enhanced the proportion of *Lactobacillus* and *Enterorhabdus*, while slightly increasing the proportion of *Lachnoclostridium*. In addition, the LBE therapy significantly enhanced the prevalence of *Bacteroides* and *Parabacteroides* while significantly reducing the prevalence of the *Ruminococcaceae NK4A214 group*, although there was no significant difference in these genera between the SOD1^G93A^ and WT mice (Fig. 9d and e).

### Effect of LBE Treatment on Serum SCFAs in SOD1^G93A^ Mice

**Fig. 10:**
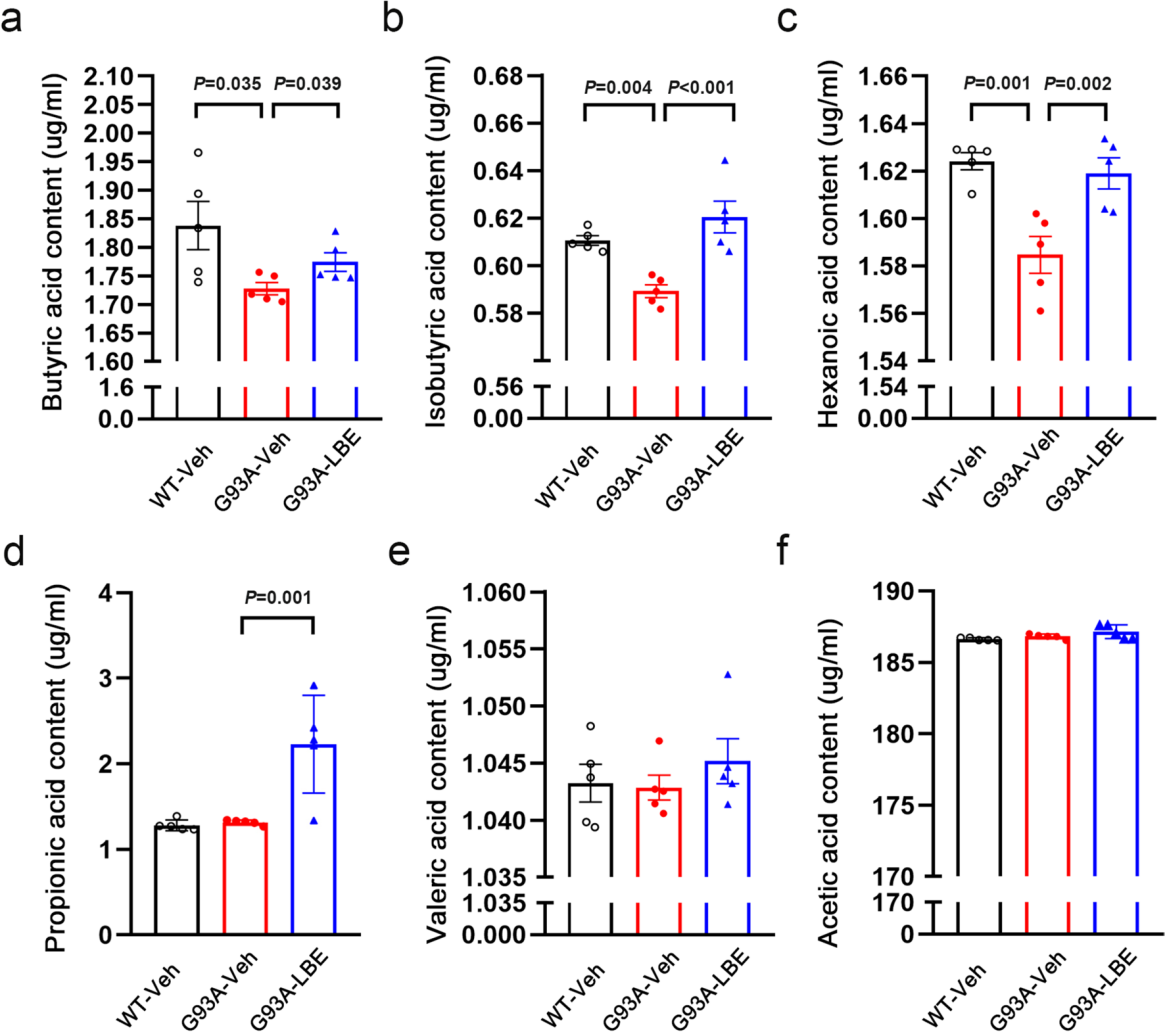
Effect of LBE on serum SCFA levels in SOD1^G93A^ mice. Serum levels of butyric acid (**a**), isobutyric acid (**b**), hexanoic acid (**c**), propionic acid (**d**), valeric acid (**e**), and acetic acid (**f**) among different groups (WT-Veh, G93A-Veh, and G93A-LBE mice). *n* = 5 mice per group

Intestinal microbiota ferments dietary fiber to produce SCFAs, which are believed to have a mediating function in interactions between microbiota, the gut, and the CNS [47]. Decreased levels of SCFAs have been observed in both patients and animal models of various neurodegenerative disorders [19, 48]. Consequently, we analyzed the levels of SCFAs in the serum of the experimental cohorts. In the SOD1^G93A^ mice, there was a reduction in serum levels of butyric acid, isobutyric acid, and hexanoic acid, as opposed to the WT control mice. However, treatment with LBE reversed this decrease and restored the concentrations of butyric acid, isobutyric acid, and hexanoic acid in the SOD1^G93A^ mice (Fig. 10a–c). Furthermore, LBE therapy markedly elevated the levels of propionic acid in the serum (Fig. 10d). Nevertheless, the concentrations of valeric acid and acetic acid showed no variations across the three groups (Fig. 10e and f). The results suggested that LBE-mediated pathophysiological improvement in the intestine and spinal cord could be attributed to increased SCFA levels.

### LBE Enhanced the Level of Autophagy and Promoted the Degradation of Abnormal SOD1 Protein in SOD1^G93A^ Mice

**Fig. 11:**
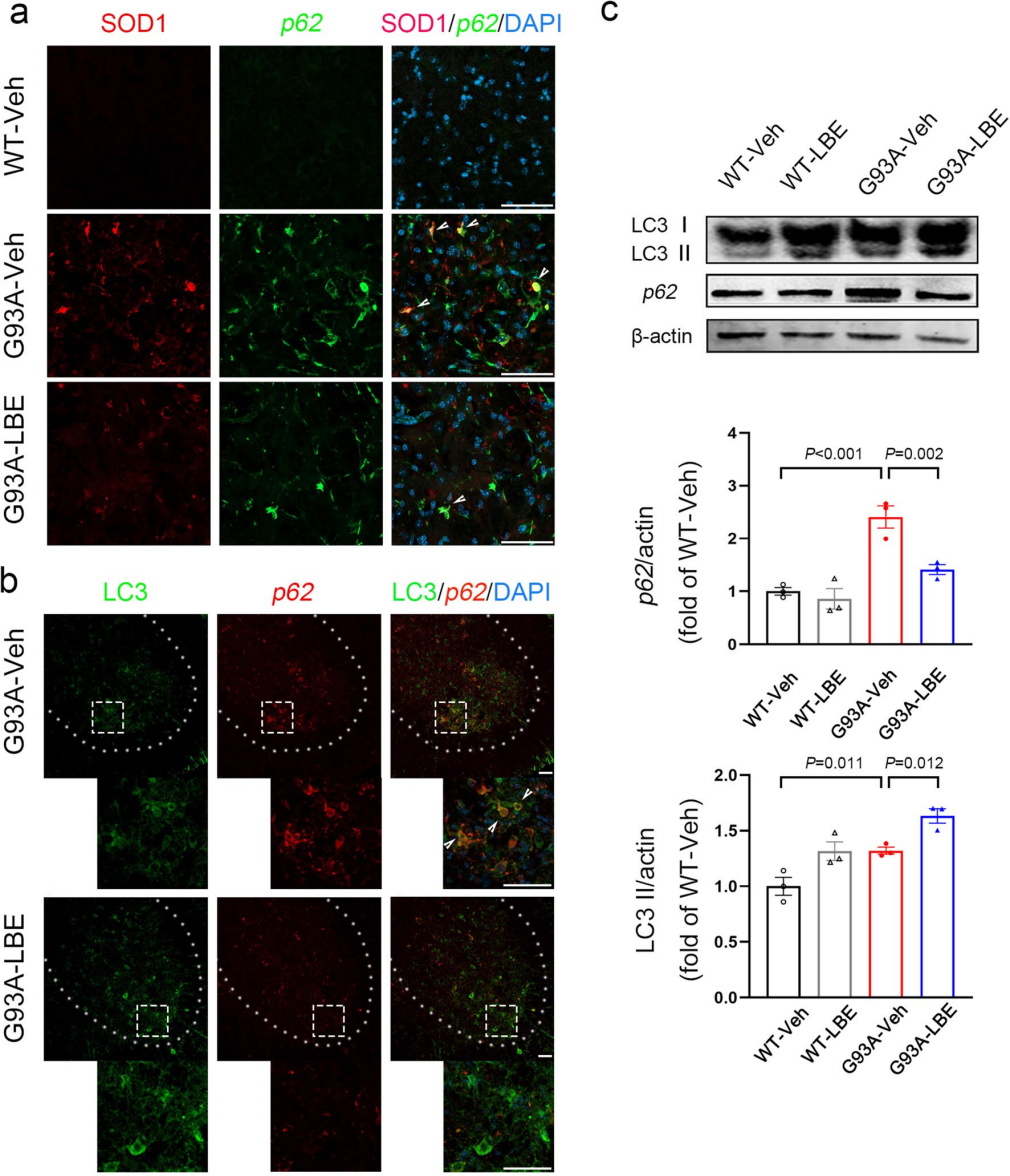
LBE enhanced the autophagy level and promoted the degradation of abnormal SOD1 proteins in SOD1^G93A^ mice. **a** Co-immunofluorescence staining of hSOD1 (red) and p62 (green) in the lumbar spinal cord of mice in all groups (WT-Veh, G93A-Veh, and G93A-LBE mice) at 120 days of age. White arrowheads show the colocalizations of hSOD1 ^+^ and p62 ^+^ . Scale bar = 50 μm. **b** Co-immunofluorescence staining of p62 (red) and LC3 (green) in the lumbar spinal cord of mice in all groups (G93A-Veh and G93A-LBE mice) at 120 days of age. White arrowheads show the colocalizations of p62 ^+^ and LC3 ^+^ . Scale bar = 50 μm. **c** WB analysis of p62 and LC3 levels in the lumbar spinal cord of mice in all groups (WT-Veh, WT-LBE, G93A-Veh, and G93A-LBE mice) at 120 days of age. Quantification of LC3II and p62 relative to actin. *n* = 3 mice per group. Nuclei were stained with DAPI (blue)

In order to investigate the potential mechanism through which LBE decreases abnormal SOD1 proteins in SOD1^G93A^ mice, we assessed the impact of LBE on autophagy in SOD1^G93A^ mice. The reason for choosing this mechanism is that in eukaryotic cells, the autophagy-lysosomal pathway is primarily responsible for the degradation of abnormal protein in the cytoplasm [49]. Additionally, autophagy regulation is linked to intestinal microbiota and their metabolites, including SCFAs [8, 26, 50]. Recent research has indicated that autophagy-specific substrates, like sequestosome-1 (SQSTM1/p62), attach to distinct substances and enter autophagosomes, which subsequently merge with lysosomes for the degradation of the proteins [49]. Consequently, we investigated the p62 expression in the lumbar spinal cord and its correlation with abnormal SOD1 proteins. Using immunofluorescence staining, we detected extensive p62 aggregations in SOD1^G93A^ mice, which were found to colocalize with abnormal SOD1 proteins (Fig. 11a), indicating that abnormal autophagy played a role in the accumulation of abnormal SOD1 proteins in SOD1^G93A^ mice. The colocalizations were significantly reduced by LBE treatment (Fig. 11a), indicating that the utilization of LBE could potentially have a beneficial effect by promoting autophagy and facilitating the breakdown of abnormal SOD1 proteins. In order to demonstrate this assertion, we conducted experiments to examine the levels of LC3 and p62 in the lumbar spinal cord using immunofluorescence and WB techniques. In SOD1^G93A^ mice, it was observed that the levels of p62 and LC3 were considerably elevated compared to the WT mice. Additionally, there were obvious colocalizations of both in SOD1^G93A^ mice (Fig. 11b and c), indicating abnormal autophagic flux in the SOD1^G93A^ mice impaired the ability of autophagy to clear abnormal proteins. Nevertheless, LBE therapy notably enhanced LC3 expression while reducing p62 expression (Fig. 11b and c), indicating that LBE treatment enhanced autophagy and promoted autophagic flux, thereby facilitating the degradation of abnormal SOD1 proteins in SOD1^G93A^ mice.

## Discussion

The current study presented new evidence indicating the existence of intestinal structural abnormalities throughout the progression of ALS disease. We found pathological SOD1 proteins aggregated in the myenteric neurons of SOD1^G93A^ mice. This discovery appears credible as the ENS shares comparable domain structures with the CNS. Therefore, the pathogenic mechanisms whereby CNS disease may lead to damage and dysfunction of the ENS. This perspective is backed by multiple studies on the gastrointestinal structure of CNS disorders [9]. The present study also found that myenteric neuron loss was accompanied by increased expression of enteric glial cells surrounding myenteric neurons and inflammatory cell proliferation in SOD1^G93A^ mice. As it is unclear whether these pathological changes affect the disease phenotypes of ALS, we speculate that these changes might be involved in abnormal intestinal motility, gut microbial dysbiosis, and abnormal immune function in SOD1^G93A^ mice based on their complex biological functions [9, 51].

In line with previous findings [14], our results revealed a compromised integrity of the intestinal epithelial barrier in SOD1^G93A^ mice. Nevertheless, the cause of this remains uncertain. We speculate that it might be derived from multiple factors, as there is a strong association between the intestinal epithelial barrier, the ENS, the intestinal microbiome, and their metabolites [9, 52]. The intestinal mucus barrier protects its host from harmful stimuli invasions and contributes to intestinal homeostasis. Damage to the barrier can lead to leakage of some harmful constituents, like bacterial endotoxins. Activation of the toll-like receptor 4/nuclear factor-kappa B (NF-κB) signaling pathway by endotoxins can result in an upsurge in the production of proinflammatory cytokines, including TNF-α and IL-1β, which ultimately disrupts the balance of the inflammatory response [53]. The outcome aligns with our findings from the SOD1^G93A^ mouse model, indicating an elevated presence of proinflammatory cells in the colon. Nonetheless, we observed no notable decline in tight junctions or a rise in submucosal inflammatory cells in the ileum with less microbiota, confirming the involvement of intestinal microbiota in the aforementioned pathological alterations in SOD1^G93A^ mice. Moreover, certain reports suggest that intestinal dysbiosis destroys the integrity of the intestinal barrier, resulting in the infiltration of harmful pathogens and toxic byproducts into the organism. As a result, prolonged inflammation causes the buildup of toxic misfolded proteins inside and outside of cells due to impaired immune response, resulting in cellular demise [8]. This theory could potentially elucidate the limited presence of abnormal SOD1 aggregates in the ileum of SOD1^G93A^ mice, in contrast to the colon.

Probiotics can regulate the intestinal microbiota, affect the pathophysiology of the gut, and provide positive effects on the CNS via the “microbiota-gut-brain” axis [40, 44, 54, 55]. The current investigation demonstrated that there was a variation in the composition of gut microbiota between SOD1^G93A^ mice and their WT littermates. We observed a notable decline in the ratio of Firmicutes/Proteobacteria. Previous studies have indicated that Firmicutes were associated with motor improvement in spinal cord injury and contributed to butyrate and propionate synthesis [13, 56]. Nonetheless, a notably increased prevalence of Proteobacteria was linked to malfunction of the intestinal epithelial cells [57] and was detected in cases of colitis and certain neurological disorders [58–60]. At the genus level, a decline in the prevalence of *Lactobacillus* was observed in mice with the SOD1^G93A^ mutation. These bacteria are generally considered beneficial microbiota [23]. Numerous researches have proven that *Lactobacillus* can influence mucosal barrier function, modulate the immune system, and effectively hinder the growth and proliferation of harmful bacteria [23, 61]. *Lactobacillus* has been recognized as a creator of SCFAs [62], and its abundance in intestinal microbiota is related to an improvement in various neurological disease symptoms [40, 42, 63]. Furthermore, the SOD1^G93A^ mice exhibited a reduction in the prevalence of *Lachnoclostridium* and *Enterorhabdus*. *Lachnoclostridium* levels were also related to the production of SCFAs [64]. The results indicate that an imbalance in the gut microbiota, specifically a reduction in bacteria that produce SCFAs, could play a role in the intestinal damage associated with ALS. Promisingly, the LBE administration effectively regulated the gut microecology and partially rectified the imbalanced gut microbiota in SOD1^G93A^ mice. Moreover, it was discovered that the application of LBE enhanced the manifestation of tight junction proteins in the intestines, decreased abnormal protein aggregation, and mitigated proinflammatory reactions in the spinal cord and gut. There might be a potential correlation between the gut microbiome and the aforementioned pathological alterations. However, it is important to note that previous studies on the microflora of ALS animal model and patients have obtained contradictory results [65]. One major reason for this phenomenon is that the gut microbiota is affected by a variety of external environment, including host age, heredity, diet, and exercise [66, 67]. A study on ALS patients shows that probiotic supplementation does not significant alter the fecal microbiota composition indicators [68]. This apparent discrepancy in the results might be related to the oral dose and type of probiotics, duration of treatment, differences in samples (fecal or cecal samples), and environmental factors may affect the composition of gut microbiota.

Probiotic-mediated pathology improvement may originate from many factors. In the present study, LBE contributed to elevated SCFA concentrations. Their contribution is vital for preserving the integrity of the gut barrier [69]. Furthermore, they can modulate the immune system and inflammation by decreasing the recruitment of multiple inflammatory cells, as well as controlling the generation and release of various cytokines like TNF-α, IL-1, and IL-10 [17, 70]. Recent studies have demonstrated that SCFAs regulate microglia and neuroinflammation in multiple neurological diseases [10, 71, 72]. Additionally, these metabolites may decrease neurotoxic misfolded protein accumulations triggered by chronic inflammation and thus positively affect ALS. Notably, prior research has discovered the accumulations of p62 in the spinal cord of both individuals and ALS animal models, indicating that dysregulation of the autophagy-lysosomal pathway may be a common feature of this disease [6, 73]. The confirmed protective role of activated autophagy on the reduction of toxic protein aggregates has been confirmed in ALS and various neurodegenerative diseases [74–76]. In this study, LBE treatment enhanced the level of autophagy and promoted the degradation of the autophagy substrate proteins and abnormal SOD1 proteins in SOD1^G93A^ mice. This effect may be due to the regulation of intestinal microbiota or increased serum SCFA levels with LBE treatment. As found by Lin et al. [26], *Bifidobacterium* treatment can induce autophagy activation in epithelial cells of the gut; however, the potential mechanism behind this phenomenon remains uninvestigated. Intriguingly, SCFAs also exhibited regulatory impacts on autophagy. Research has discovered that in skeletal muscle cells of diabetic nephropathy and colon cancer cells, butyrate can exert an influence on autophagy and is correlated with mammalian-target-of-rapamycin mTOR pathway alterations [50, 77]. Furthermore, SCFAs are involved in regulating the activity of histone deacetylases (HDACs), which are related to autophagy regulation [78, 79]. Thus, the regulation of autophagy could potentially be the primary factor behind the reduction in abnormal SOD1 accumulations observed in the intestine and spinal cord following LBE therapy.

**Fig. 12:**
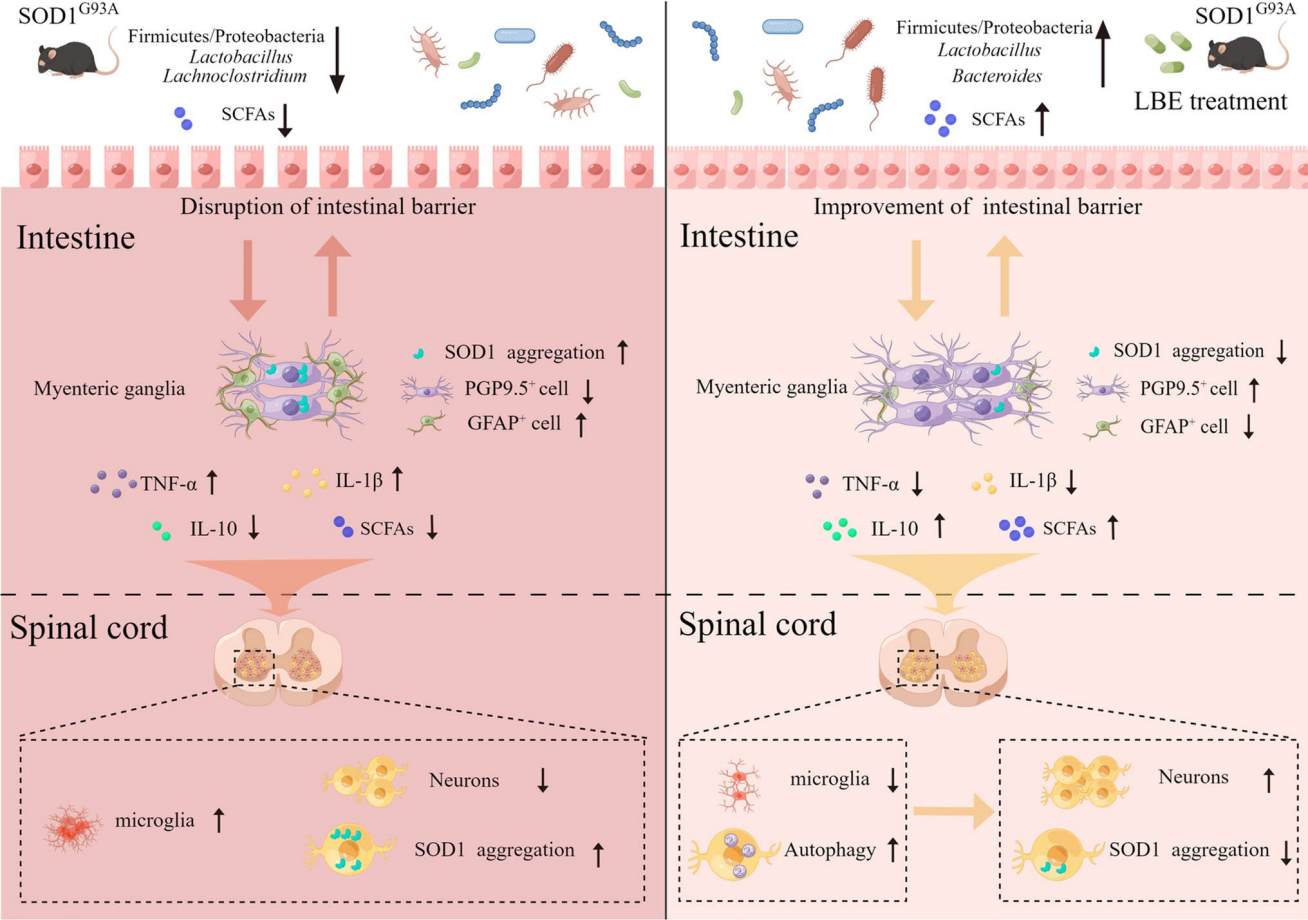
Intestinal microbiota dysbiosis and intestinal injury in SOD1^G93A^ mice and working mechanism diagram of LBE treatment

The intestine serves as a conduit for interaction between microbes and the brain. Multiple studies have reported that mucosal barrier function is related to the immunoregulatory effects of intestinal microbiota on various nervous system diseases [11, 12]. This could be connected to its crucial function in preventing the passage of immune stimulators, such as peptidoglycans and lipopolysaccharides, from the lumen to the bloodstream. Furthermore, earlier research has suggested that proinflammatory substances originating from the gut could potentially enter the brain via the bloodstream and impact neuronal homeostasis [12, 13]. This implies that the gut microbiota might indirectly alter the central nervous system’s disease progression in ALS by modifying the homeostasis of the intestine. The mechanism schematic is given in Fig. 12 summarized from the previous analysis.

Nevertheless, despite the positive results achieved in SOD1^G93A^ mice, the precise mechanism underlying the advantageous impact of LBE is complex and requires clarification. Additional investigation is necessary to establish conclusive findings and develop a comprehensive approach for treating ALS.

## Conclusion

To summarize, our investigation presented new evidence of abnormal intestinal structure in SOD1^G93A^ mice. We observed the accumulation of pathological SOD1 in the myenteric neurons of SOD1^G93A^ mice. The presence of this pathological occurrence was accompanied by ENS structure abnormalities, impaired barrier function, and an excessively activated proinflammatory reaction in the intestine. The presence of distinct pathological variances in the colon and ileum indicates a potential association with the luminal microbiota. LBE has the potential to improve the proinflammatory response, decrease aberrant SOD1 aggregation, and protect neuronal cells in the spinal cord and intestine. The positive effect of LBE can be attributed to increased SCFAs and enhanced autophagy flux.

## Supplementary Information

Below is the link to the electronic supplementary material.

Supplementary file1 (DOCX 892 KB)

## Data Availability

The raw data supporting the conclusions of this article will be made available by the authors, without undue reservation.

## References

[1] Brown RH, Al-Chalabi A (2017) Amyotrophic lateral sclerosis. N Engl J Med 377:162–172 28700839 10.1056/NEJMra1603471

[2] Rosen DR (1993) Mutations in Cu/Zn superoxide dismutase gene are associated with familial amyotrophic lateral sclerosis. Nature 364:362 8332197 10.1038/364362c0

[3] Li D, Liu C (2022) Conformational strains of pathogenic amyloid proteins in neurodegenerative diseases. Nat Rev Neurosci 23:523–534 35637417 10.1038/s41583-022-00603-7

[4] Mackenzie IR, Neumann M (2012) FET proteins in frontotemporal dementia and amyotrophic lateral sclerosis. Brain Res 1462:40–43 22261247 10.1016/j.brainres.2011.12.010

[5] Jawaid A, Khan R, Polymenidou M, Schulz PE (2018) Disease-modifying effects of metabolic perturbations in ALS/FTLD. Mol Neurodegener 13:63 30509290 10.1186/s13024-018-0294-0 PMC6278047

[6] Rudnick ND, Griffey CJ, Guarnieri P, Gerbino V, Wang X, Piersaint JA et al (2017) Distinct roles for motor neuron autophagy early and late in the SOD1(G93A) mouse model of ALS. Proc Natl Acad Sci U S A 114:E8294–E8303 28904095 10.1073/pnas.1704294114 PMC5625902

[7] Staats KA, Borchelt DR, Tansey MG, Wymer J (2022) Blood-based biomarkers of inflammation in amyotrophic lateral sclerosis. Mol Neurodegener 17:11 35073950 10.1186/s13024-022-00515-1 PMC8785449

[8] Chidambaram SB, Essa MM, Rathipriya AG, Bishir M, Ray B, Mahalakshmi AM et al (2022) Gut dysbiosis, defective autophagy and altered immune responses in neurodegenerative diseases: tales of a vicious cycle. Pharmacol Ther 231:107988 34536490 10.1016/j.pharmthera.2021.107988

[9] Niesler B, Kuerten S, Demir IE, Schafer KH (2021) Disorders of the enteric nervous system - a holistic view. Nat Rev Gastroenterol Hepatol 18:393–410 33514916 10.1038/s41575-020-00385-2

[10] Li JM, Yu R, Zhang LP, Wen SY, Wang SJ, Zhang XY et al (2019) Dietary fructose-induced gut dysbiosis promotes mouse hippocampal neuroinflammation: a benefit of short-chain fatty acids. Microbiome 7:98 31255176 10.1186/s40168-019-0713-7 PMC6599330

[11] Pellegrini C, Antonioli L, Colucci R, Blandizzi C, Fornai M (2018) Interplay among gut microbiota, intestinal mucosal barrier and enteric neuro-immune system: a common path to neurodegenerative diseases? Acta Neuropathol 136:345–361 29797112 10.1007/s00401-018-1856-5

[12] Serra D, Almeida LM, Dinis TCP (2019) The impact of chronic intestinal inflammation on brain disorders: the microbiota-gut-brain axis. Mol Neurobiol 56:6941–6951 30945157 10.1007/s12035-019-1572-8

[13] Jing Y, Yu Y, Bai F, Wang L, Yang D, Zhang C et al (2021) Effect of fecal microbiota transplantation on neurological restoration in a spinal cord injury mouse model: involvement of brain-gut axis. Microbiome 9:59 33678185 10.1186/s40168-021-01007-y PMC7937282

[14] Wu S, Yi J, Zhang YG, Zhou J, Sun J (2015) Leaky intestine and impaired microbiome in an amyotrophic lateral sclerosis mouse model. Physiol Rep 3:e12356 25847918 10.14814/phy2.12356 PMC4425962

[15] Sender R, Fuchs S, Milo R (2016) Revised estimates for the number of human and bacteria cells in the body. PLoS Biol 14:e1002533 27541692 10.1371/journal.pbio.1002533 PMC4991899

[16] Cryan JF, O’Riordan KJ, Sandhu K, Peterson V, Dinan TG (2020) The gut microbiome in neurological disorders. Lancet Neurol 19:179–194 31753762 10.1016/S1474-4422(19)30356-4

[17] Singh N, Singh V, Rai SN, Mishra V, Vamanu E, Singh MP (2022) Deciphering the gut microbiome in neurodegenerative diseases and metagenomic approaches for characterization of gut microbes. Biomed Pharmacother 156:113958 36411639 10.1016/j.biopha.2022.113958

[18] Sampson TR, Debelius JW, Thron T, Janssen S, Shastri GG, Ilhan ZE et al (2016) Gut microbiota regulate motor deficits and neuroinflammation in a model of Parkinson’s disease. Cell 167:1469-1480.e1412 27912057 10.1016/j.cell.2016.11.018 PMC5718049

[19] Zhang YG, Wu S, Yi J, Xia Y, Jin D, Zhou J et al (2017) Target intestinal microbiota to alleviate disease progression in amyotrophic lateral sclerosis. Clin Ther 39:322–336 28129947 10.1016/j.clinthera.2016.12.014 PMC5344195

[20] Blacher E, Bashiardes S, Shapiro H, Rothschild D, Mor U, Dori-Bachash M et al (2019) Potential roles of gut microbiome and metabolites in modulating ALS in mice. Nature 572:474–480 31330533 10.1038/s41586-019-1443-5

[21] Burberry A, Wells MF, Limone F, Couto A, Smith KS, Keaney J et al (2020) C9orf72 suppresses systemic and neural inflammation induced by gut bacteria. Nature 582:89–94 32483373 10.1038/s41586-020-2288-7 PMC7416879

[22] Gibson GR, Hutkins R, Sanders ME, Prescott SL, Reimer RA, Salminen SJ et al (2017) Expert consensus document: the International Scientific Association for Probiotics and Prebiotics (ISAPP) consensus statement on the definition and scope of prebiotics. Nat Rev Gastroenterol Hepatol 14:491–502 28611480 10.1038/nrgastro.2017.75

[23] Suissa R, Oved R, Jankelowitz G, Turjeman S, Koren O, Kolodkin-Gal I (2022) Molecular genetics for probiotic engineering: dissecting lactic acid bacteria. Trends Microbiol 30:293–306 34446338 10.1016/j.tim.2021.07.007

[24] Plaza-Diaz J, Ruiz-Ojeda FJ, Gil-Campos M, Gil A (2019) Mechanisms of action of probiotics. Adv Nutr 10:S49–S66 30721959 10.1093/advances/nmy063 PMC6363529

[25] Colquitt AS, Miles EA, Calder PC (2022) Do probiotics in pregnancy reduce allergies and asthma in infancy and childhood? A systematic review Nutrients 14:1852 35565819 10.3390/nu14091852 PMC9105059

[26] Lin R, Jiang Y, Zhao XY, Guan Y, Qian W, Fu XC et al (2014) Four types of *Bifidobacteria* trigger autophagy response in intestinal epithelial cells. J Dig Dis 15:597–605 25123057 10.1111/1751-2980.12179

[27] Srivastav S, Neupane S, Bhurtel S, Katila N, Maharjan S, Choi H et al (2019) Probiotics mixture increases butyrate, and subsequently rescues the nigral dopaminergic neurons from MPTP and rotenone-induced neurotoxicity. J Nutr Biochem 69:73–86 31063918 10.1016/j.jnutbio.2019.03.021

[28] Sun H, Zhao F, Liu Y, Ma T, Jin H, Quan K et al (2022) Probiotics synergized with conventional regimen in managing Parkinson’s disease. NPJ Parkinsons Dis 8:62 35610236 10.1038/s41531-022-00327-6 PMC9130297

[29] Azad MAK, Sarker M, Li T, Yin J (2018) Probiotic species in the modulation of gut microbiota: an overview. Biomed Res Int 2018:9478630 29854813 10.1155/2018/9478630 PMC5964481

[30] de Vos WM, Tilg H, Van Hul M, Cani PD (2022) Gut microbiome and health: mechanistic insights. Gut 71:1020–1032 35105664 10.1136/gutjnl-2021-326789 PMC8995832

[31] de Rijke TJ, Doting MHE, van Hemert S, De Deyn PP, van Munster BC, Harmsen HJM et al (2022) A systematic review on the effects of different types of probiotics in animal Alzheimer’s disease studies. Front Psychiatr 13:879491 10.3389/fpsyt.2022.879491 PMC9094066 35573324

[32] Sarao LK, Arora M (2015) Probiotics, prebiotics, and microencapsulation: a review. Crit Rev Food Sci Nutr 57:344–371 10.1080/10408398.2014.887055 25848935

[33] Azad MAK, Sarker M, Li T, Yin J (2018) Probiotic species in the modulation of gut microbiota: an overview. Biomed Res Int 2018:1–8 10.1155/2018/9478630 PMC5964481 29854813

[34] Tomasz B, Zoran S, Jarosław W, Ryszard M, Marcin G, Robert B et al (2014) Long-term use of probiotics Lactobacillus and Bifidobacterium has a prophylactic effect on the occurrence and severity of pouchitis: a randomized prospective study. Biomed Res Int 2014:1–4 10.1155/2014/208064 PMC3918689 24579075

[35] Plaza-Diaz J, Ruiz-Ojeda FJ, Gil-Campos M, Gil A (2019) Mechanisms of action of probiotics. Adv Nutr 10:S49–S66 30721959 10.1093/advances/nmy063 PMC6363529

[36] Sugahara H, Odamaki T, Fukuda S, Kato T, Xiao J-z, Abe F et al (2015) Probiotic Bifidobacterium longum alters gut luminal metabolism through modification of the gut microbial community. Sci Rep 5:13548 10.1038/srep13548 PMC4552000 26315217

[37] Chen Y-M, Li Y, Wang X, Wang Z-L, Hou J-J, Su S et al (2022) Effect of Bacillus subtilis, Enterococcus faecium, and Enterococcus faecalis supernatants on serotonin transporter expression in cells and tissues. World J Gastroenterol 28:532–546 35316963 10.3748/wjg.v28.i5.532 PMC8905020

[38] Nami Y, Haghshenas B, Yari KA (2018) Molecular identification and probiotic potential characterization of lactic acid bacteria isolated from human vaginal microbiota. Adv Pharm Bull 8:683–695 30607341 10.15171/apb.2018.077 PMC6311637

[39] Zischka M, Künne CT, Blom J, Wobser D, Sakιnç T, Schmidt-Hohagen et al (2015) Comprehensive molecular, genomic and phenotypic analysis of a major clone of Enterococcus faecalis MLST ST40. BMC Genomics 16:175 10.1186/s12864-015-1367-x PMC4374294 25887115

[40] Lee HJ, Hwang YH, Kim DH (2018) *Lactobacillus**plantarum* C29-fermented soybean (DW2009) alleviates memory impairment in 5XFAD transgenic mice by regulating microglia activation and gut microbiota composition. Mol Nutr Food Res 62:e1800359 30152045 10.1002/mnfr.201800359

[41] Kobayashi Y, Sugahara H, Shimada K, Mitsuyama E, Kuhara T, Yasuoka A et al (2017) Therapeutic potential of Bifidobacterium breve strain A1 for preventing cognitive impairment in Alzheimer’s disease. Sci Rep 7:13510 29044140 10.1038/s41598-017-13368-2 PMC5647431

[42] Liao JF, Cheng YF, You ST, Kuo WC, Huang CW, Chiou JJ et al (2020) *Lactobacillus**plantarum* PS128 alleviates neurodegenerative progression in 1-methyl-4-phenyl-1,2,3,6-tetrahydropyridine-induced mouse models of Parkinson’s disease. Brain Behav Immun 90:26–46 32739365 10.1016/j.bbi.2020.07.036

[43] Athari Nik Azm S, Djazayeri A, Safa M, Azami K, Ahmadvand B, Sabbaghziarani F et al (2018) Lactobacilli and bifidobacteria ameliorate memory and learning deficits and oxidative stress in beta-amyloid (1–42) injected rats. Appl Physiol Nutr Metab 43:718–726 29462572 10.1139/apnm-2017-0648

[44] Vamanu E, Rai SN (2021) The link between obesity, microbiota dysbiosis, and neurodegenerative pathogenesis. Diseases 9:45 34201465 10.3390/diseases9030045 PMC8293145

[45] Wang Y, Bai L, Li S, Wen Y, Liu Q, Li R et al (2021) Simvastatin enhances muscle regeneration through autophagic defect-mediated inflammation and mTOR activation in G93ASOD1 mice. Mol Neurobiol 58:1593–1606 33222146 10.1007/s12035-020-02216-6

[46] Hatzipetros T, Kidd JD, Moreno AJ, Thompson K, Gill A, Vieira FG (2015) A quick phenotypic neurological scoring system for evaluating disease progression in the SOD1-G93A mouse model of ALS. J Vis Exp 53257:1–6 10.3791/53257 PMC4692639 26485052

[47] Dalile B, Van Oudenhove L, Vervliet B, Verbeke K (2019) The role of short-chain fatty acids in microbiota-gut-brain communication. Nat Rev Gastroenterol Hepatol 16:461–478 31123355 10.1038/s41575-019-0157-3

[48] Chen SJ, Chen CC, Liao HY, Lin YT, Wu YW, Liou JM et al (2022) Association of fecal and plasma levels of short-chain fatty acids with gut microbiota and clinical severity in patients with Parkinson disease. Neurology 98:e848–e858 34996879 10.1212/WNL.0000000000013225 PMC8883514

[49] Pohl C, Dikic I (2019) Cellular quality control by the ubiquitin-proteasome system and autophagy. Science 366:818–822 31727826 10.1126/science.aax3769

[50] Tang Y, Chen Y, Jiang H, Nie D (2011) Short-chain fatty acids induced autophagy serves as an adaptive strategy for retarding mitochondria-mediated apoptotic cell death. Cell Death Differ 18:602–618 20930850 10.1038/cdd.2010.117 PMC3020988

[51] Progatzky F, Shapiro M, Chng SH, Garcia-Cassani B, Classon CH, Sevgi S et al (2021) Regulation of intestinal immunity and tissue repair by enteric glia. Nature 599:125–130 34671159 10.1038/s41586-021-04006-z PMC7612231

[52] Wu WH, Kim M, Chang LC, Assie A, Saldana-Morales FB, Zegarra-Ruiz DF et al (2022) Interleukin-1beta secretion induced by mucosa-associated gut commensal bacteria promotes intestinal barrier repair. Gut Microbes 14:2014772 34989321 10.1080/19490976.2021.2014772 PMC8741296

[53] Bryant CE, Spring DR, Gangloff M, Gay NJ (2010) The molecular basis of the host response to lipopolysaccharide. Nat Rev Microbiol 8:8–14 19946286 10.1038/nrmicro2266

[54] Huang SY, Chen LH, Wang MF, Hsu CC, Chan CH, Li JX et al (2018) *Lactobacillus**paracasei* PS23 delays progression of age-related cognitive decline in senescence accelerated mouse prone 8 (SAMP8) mice. Nutrients 10:894 30002347 10.3390/nu10070894 PMC6073302

[55] Liu YW, Liu WH, Wu CC, Juan YC, Wu YC, Tsai HP et al (2016) Psychotropic effects of *Lactobacillus**plantarum* PS128 in early life-stressed and naive adult mice. Brain Res 1631:1–12 26620542 10.1016/j.brainres.2015.11.018

[56] Polansky O, Sekelova Z, Faldynova M, Sebkova A, Sisak F, Rychlik I (2015) Important metabolic pathways and biological processes expressed by chicken cecal microbiota. Appl Environ Microbiol 82:1569–1576 26712550 10.1128/AEM.03473-15 PMC4771310

[57] Litvak Y, Byndloss MX, Tsolis RM, Baumler AJ (2017) Dysbiotic Proteobacteria expansion: a microbial signature of epithelial dysfunction. Curr Opin Microbiol 39:1–6 28783509 10.1016/j.mib.2017.07.003

[58] Shi H, Ge X, Ma X, Zheng M, Cui X, Pan W et al (2021) A fiber-deprived diet causes cognitive impairment and hippocampal microglia-mediated synaptic loss through the gut microbiota and metabolites. Microbiome 9:223 34758889 10.1186/s40168-021-01172-0 PMC8582174

[59] Gobert AP, Latour YL, Asim M, Barry DP, Allaman MM, Finley JL et al (2022) Protective role of spermidine in colitis and colon carcinogenesis. Gastroenterology 162:813-827.e818 34767785 10.1053/j.gastro.2021.11.005 PMC8881368

[60] Xu R, Tan C, He Y, Wu Q, Wang H, Yin J (2020) Dysbiosis of gut microbiota and short-chain fatty acids in encephalitis: a Chinese pilot study. Front Immunol 11:1994 32973805 10.3389/fimmu.2020.01994 PMC7468513

[61] Di Cerbo A, Palmieri B, Aponte M, Morales-Medina JC, Iannitti T (2016) Mechanisms and therapeutic effectiveness of lactobacilli. J Clin Pathol 69:187–203 26578541 10.1136/jclinpath-2015-202976 PMC4789713

[62] Nyangale EP, Mottram DS, Gibson GR (2012) Gut microbial activity, implications for health and disease: the potential role of metabolite analysis. J Proteome Res 11:5573–5585 23116228 10.1021/pr300637d

[63] Jeon S, Kim H, Kim J, Seol D, Jo J, Choi Y et al (2022) Positive effect of *Lactobacillus**acidophilus* EG004 on cognitive ability of healthy mice by fecal microbiome analysis using full-length 16S–23S rRNA metagenome sequencing. Microbiol Spectr 10:e0181521 35019699 10.1128/spectrum.01815-21 PMC8754107

[64] Chen L, Zhou X, Wang Y, Wang D, Ke Y, Zeng X (2021) Propionate and butyrate produced by gut microbiota after probiotic supplementation attenuate lung metastasis of melanoma cells in mice. Mol Nutr Food Res 65:e2100096 34061433 10.1002/mnfr.202100096

[65] Sun J, Huang T, Debelius JW, Fang F (2021) Gut microbiome and amyotrophic lateral sclerosis: a systematic review of current evidence. J Intern Med 290:758–788 34080741 10.1111/joim.13336

[66] Zheng X, Huang F, Zhao A, Lei S, Zhang Y, Xie G et al (2015) Bile acid is a significant host factor shaping the gut microbiome of diet-induced obese mice. BMC Biol 15:120 10.1186/s12915-017-0462-7 PMC5731064 29241453

[67] Zhang M, Tang H, Chen Y, Chen Z, Xu Y, Fu X et al (2023) Impact of environmental characteristics on children’s gut microbiota - a pilot study in assessing the role of indoor microbiome and metabolites. Environ Res 234:116114 37209986 10.1016/j.envres.2023.116114

[68] Di Gioia D, Bozzi Cionci N, Baffoni L, Amoruso A, Pane M, Mogna L et al (2020) A prospective longitudinal study on the microbiota composition in amyotrophic lateral sclerosis. BMC Med 18:153 10.1186/s12916-020-01607-9 PMC7298784 32546239

[69] Feng Y, Wang Y, Wang P, Huang Y, Wang F (2018) Short-chain fatty acids manifest stimulative and protective effects on intestinal barrier function through the inhibition of NLRP3 inflammasome and autophagy. Cell Physiol Biochem 49:190–205 30138914 10.1159/000492853

[70] Yao Y, Cai X, Fei W, Ye Y, Zhao M, Zheng C (2022) The role of short-chain fatty acids in immunity, inflammation and metabolism. Crit Rev Food Sci Nutr 62:1–12 33261516 10.1080/10408398.2020.1854675

[71] Hou Y, Li X, Liu C, Zhang M, Zhang X, Ge S et al (2021) Neuroprotective effects of short-chain fatty acids in MPTP induced mice model of Parkinson’s disease. Exp Gerontol 150:111376 33905875 10.1016/j.exger.2021.111376

[72] Erny D, Hrabe de Angelis AL, Jaitin D, Wieghofer P, Staszewski O, David E et al (2015) Host microbiota constantly control maturation and function of microglia in the CNS. Nat Neurosci 18:965–977 26030851 10.1038/nn.4030 PMC5528863

[73] Chua JP, De Calbiac H, Kabashi E, Barmada SJ (2022) Autophagy and ALS: mechanistic insights and therapeutic implications. Autophagy 18:254–282 34057020 10.1080/15548627.2021.1926656 PMC8942428

[74] Zhang X, Chen S, Song L, Tang Y, Shen Y, Jia L et al (2014) MTOR-independent, autophagic enhancer trehalose prolongs motor neuron survival and ameliorates the autophagic flux defect in a mouse model of amyotrophic lateral sclerosis. Autophagy 10:588–602 24441414 10.4161/auto.27710 PMC4091147

[75] Hetz C, Thielen P, Matus S, Nassif M, Court F, Kiffin R et al (2009) XBP-1 deficiency in the nervous system protects against amyotrophic lateral sclerosis by increasing autophagy. Genes Dev 23:2294–2306 19762508 10.1101/gad.1830709 PMC2758741

[76] Dou C, Zhang Y, Zhang L, Qin C (2023) Autophagy and autophagy-related molecules in neurodegenerative diseases. Animal Model Exp Med 6:10–17 35730702 10.1002/ame2.12229 PMC9986236

[77] Tang G, Du Y, Guan H, Jia J, Zhu N, Shi Y et al (2022) Butyrate ameliorates skeletal muscle atrophy in diabetic nephropathy by enhancing gut barrier function and FFA2-mediated PI3K/Akt/mTOR signals. Br J Pharmacol 179:159–178 34638162 10.1111/bph.15693

[78] Son SM, Park SJ, Fernandez-Estevez M, Rubinsztein DC (2021) Autophagy regulation by acetylation-implications for neurodegenerative diseases. Exp Mol Med 53:30–41 33483607 10.1038/s12276-021-00556-4 PMC8080689

[79] True O, Matthias P (2012) Interplay between histone deacetylases and autophagy–from cancer therapy to neurodegeneration. Immunol Cell Biol 90:78–84 22124372 10.1038/icb.2011.103

