# Supplementary material for: Neuroprotective Effect of a Multistrain Probiotic Mixture in SOD1G93A Mice by Reducing SOD1 Aggregation and Targeting the Microbiota-Gut-Brain Axis
Source: Mol Neurobiol. 2024 Feb 13;61(12):10051–71. doi: 10.1007/s12035-024-03988-x (PMC11584480; doi:10.1007/s12035-024-03988-x)
Supplement: Supplementary file 1 — Supplementary file1 (DOCX 892 KB) [file 12035_2024_3988_MOESM1_ESM.docx]

Supplementary Material

# Supplementary Figures


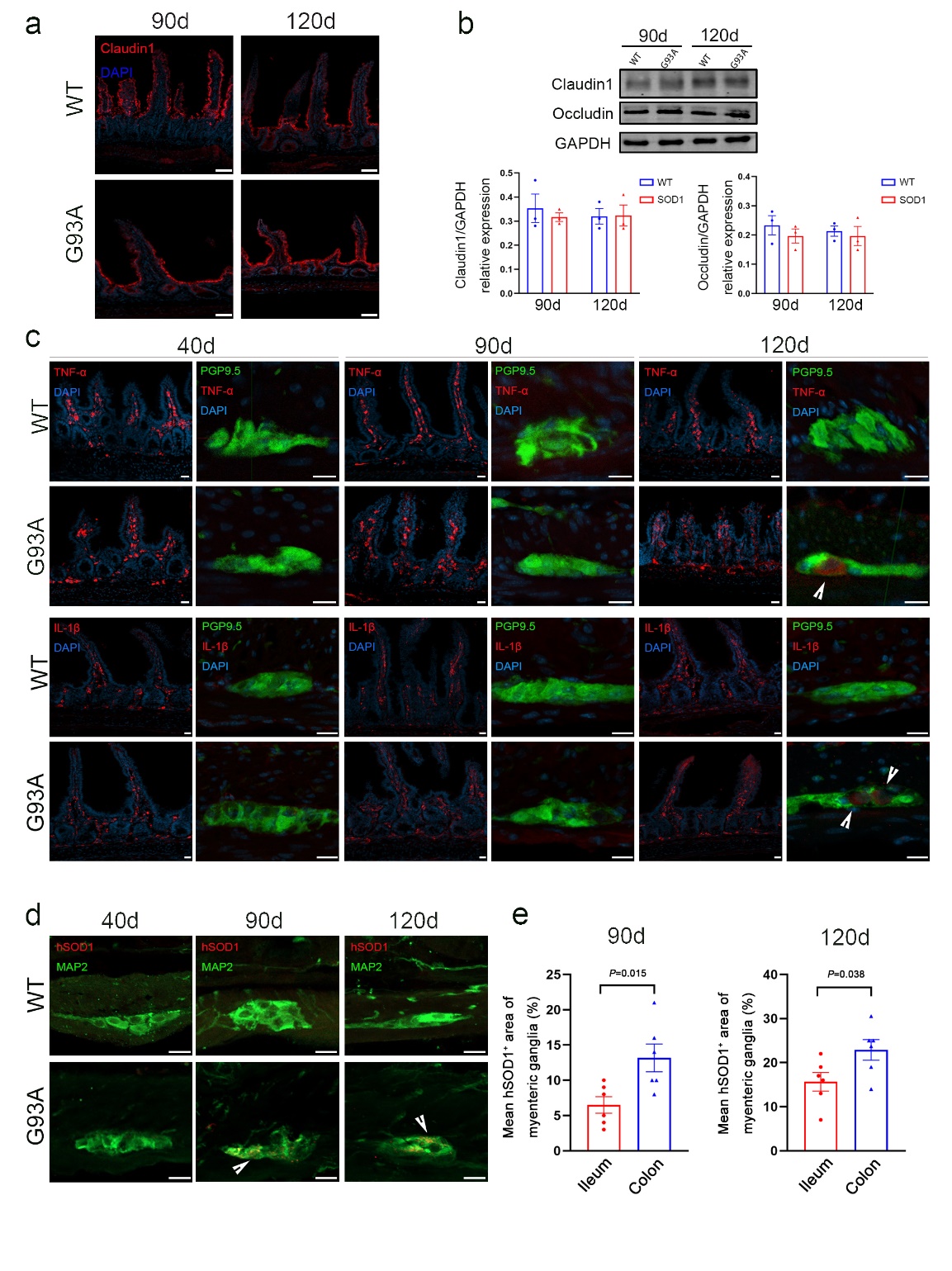


**Supplemental figure 1** The structural alterations in the ileum of SOD1^G93A^ mice. a Immunofluorescence labeling for Claudin1 (red) in the ileum of SOD1^G93A^ and WT mice at 90 days and 120 days of age. Nuclei were stained with DAPI (blue). Scale bar = 50 μm. b Western blot analysis of Claudin1 and Occludin levels in the ileum of SOD1^G93A^ and WT mice at 90 days and 120 days of age. Quantitative analysis of Claudin1 and Occludin relative to GAPDH. (Data represent the mean ± SEM, n = 3 mice per group, statistical significance was assessed by unpaired t-test). c Immunofluorescence labeling for TNF-α (red) and IL-1β (red) in the mucosa and co-immunofluorescence staining of TNF-α (red) and PGP9.5 (green), IL-1β (red) and PGP9.5 (green) in the muscle layer of ileum of SOD1G93A and WT mice at 40 days, 90 days, and 120 days of age. Nuclei were stained with DAPI (blue). White arrowheads show the portions of TNF-α+ and IL-1β+. Scale bar = 20 μm. d Co-immunofluorescence staining of hSOD1 (red) and MAP2 (green) in the muscle layer of ileum at 40 days, 90 days, and 120 days of age. White arrowheads show the portions of hSOD1+. Scale bar = 20 μm. e Comparison of mean hSOD1+ area of myenteric ganglia of ileum and colon in SOD1^G93A^ mice at 90 days and 120 days of age. (Data represent the mean ± SEM, n = 2 independent experiments with 3 mice per group, statistical significance was assessed by unpaired t-test).


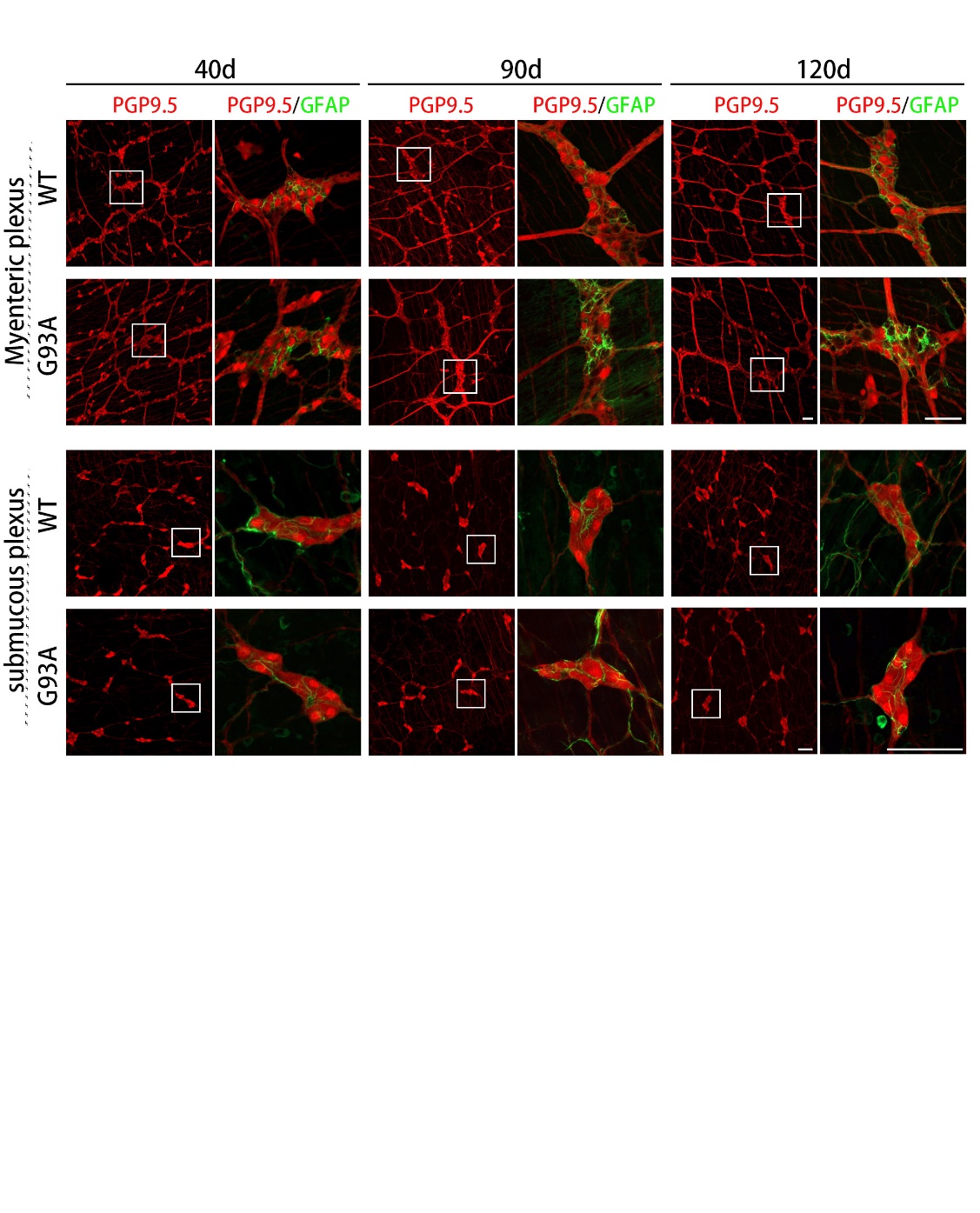


**Supplemental figure 2** Whole-mount immunofluorescence staining of the ENS showed massive loss of enteric myenteric neurons in the ileum of SOD1^G93A^ mice. Co-immunofluorescence staining of PGP9.5 (red) and GFAP (green) in the myenteric plexus and submucosal plexus of ileum of SOD1^G93A^ and WT mice at 40 days, 90 days, and 120 days of age. Scale bar = 50 μm.
